# Disease relevance of rare *VPS13B* missense variants for neurodevelopmental Cohen syndrome

**DOI:** 10.1038/s41598-022-13717-w

**Published:** 2022-06-11

**Authors:** Malte Zorn, Jirko Kühnisch, Sebastian Bachmann, Wenke Seifert

**Affiliations:** 1 Charité-Universitätsmedizin Berlin, Corporate Member of Freie Universität Berlin, Humboldt-Universität zu Berlin, and Berlin Institute of Health, Institute of Functional Anatomy, Berlin, Germany; 2 Experimental and Clinical Research Center (ECRC), a cooperation between the Max‐Delbrück‐Center for Molecular Medicine in the Helmholtz Association and the Charité‐Universitätsmedizin Berlin, Berlin, Germany; 3 Max Delbrück Center for Molecular Medicine in the Helmholtz Association (MDC), Berlin, Germany; 4 Charité-Universitätsmedizin Berlin, Corporate Member of Freie Universität Berlin, Humboldt-Universität zu Berlin, and Berlin Institute of Health, Institute of Cell Biology and Neurobiology, Charitéplatz 1, Berlin, 10117 Germany

**Keywords:** Genetics, Clinical genetics

## Abstract

Autosomal recessive Cohen syndrome is a neurodevelopmental disorder characterized by postnatal microcephaly, intellectual disability, and a typical facial gestalt. Genetic variants in *VPS13B* have been found to cause Cohen syndrome, but have also been linked to autism, retinal disease, primary immunodeficiency, and short stature. While it is well established that loss-of-function mutations of *VPS13B* cause Cohen syndrome, the relevance of missense variants for the pathomechanism remains unexplained. Here, we investigate their pathogenic effect through a systematic re-evaluation of clinical patient information, comprehensive in silico predictions, and in vitro testing of previously published missense variants. In vitro analysis of 10 subcloned *VPS13B* missense variants resulted in full-length proteins after transient overexpression. 6/10 VPS13B missense variants show reduced accumulation at the Golgi complex in the steady state. The overexpression of these 6/10 VPS13B missense variants did not rescue the Golgi fragmentation after the RNAi-mediated depletion of endogenous *VPS13B*. These results thus validate 6/10 missense variants as likely pathogenic according to the classification of the American College of Medical Genetics through the integration of clinical, genetic, in silico*,* and experimental data. In summary, we state that exact variant classification should be the first step towards elucidating the pathomechanisms of genetically inherited neuronal diseases.

## Introduction

Neurodevelopmental Cohen syndrome is caused by genetic defects in the *VPS13B* (*COH1*) gene^1,2^. Classification and functional validation of genetic disease variants is a critical challenge in clinical diagnostics and the recent explosion of annotated genetic variants strengthen the need to separate disease-related from abundant variants^3,4^. Although a vast number of prediction approaches is established, a large number of disease proteins remains to be functionally characterized^5^. In this regard, characterization of pioneer proteins without clear domain organization and proper functional analysis, such as VPS13B (COH1) is an important research topic. While Cohen syndrome is majorly caused by *VPS13B* loss-of-function mutation, a rare number of missense variants has been published to cause Cohen syndrome. These rare occurrences of disease-related missense variants in VPS13B may provide further insights on protein structure and function and may therefore facilitate the understanding of disease mechanism including genotype/phenotype correlations.

VPS13B is a large and poorly characterized disease-associated protein that is encoded by the *VPS13B* gene^6,7^. *VPS13B* localizes on chromosome 8q22 and spans a genomic region of about 864 kb comprising 62 exons^8^. The VPS13B protein is ~ 450 kDa in size (NP_689777.3, NP_060360.3) and important for Golgi structure maintenance^6^. VPS13B interacts with RAB6, RAB14, STX6, STX13, and PI3P^7,9^. Genetic variants in *VPS13B* have been linked to the neurodevelopmental disorder Cohen syndrome^2,8,10^. The autosomal recessive (AR) Cohen syndrome is characterized by intellectual disability, developmental delay, microcephaly, a characteristic facial appearance, progressive retinopathy, myopia, and/or neutropenia^2,8,11^. Up-to-date over 200 unique and rare variants in *VPS13B* have been reported to cause Cohen syndrome^1,8^. Most of these *VPS13B* variants, including nonsense, splice site, frameshift, and large exon-spanning deletions/insertions, can be classified as disease-causing due to their clearly predictable truncation of the encoded protein. Moreover, previous studies identified that premature stop mutations are most likely functional null-alleles due to nonsense-mediated mRNA decay^6,12^. Although the vast majority of *VPS13B* variants are protein length changing, 29 missense variants are reported in association with clinical conditions such as Cohen syndrome, autism, intellectual disability, retinal disease, primary immunodeficiency disease, and/or short stature^1,2,8,13–31^. However, a systematic pathogenicity assessment of these *VPS13B* missense variants has not been performed and their role for Cohen syndrome or other entities is difficult to interpret.

Previous studies showed that AR disorders are habitually caused by pathogenic variants in genes which result in loss-of-function of the encoded protein with none or little residual function. In contrast autosomal dominant (AD) disorders are caused by pathogenic variants which result equally in either loss-of, altered, or gain-of protein function^32^. Moreover, pathogenic variants most likely cluster in AD disorders to functional key regions of the encoded protein, while in AR disorders nearly no clustering of pathogenic loss-of-function variants occurs^32^. This idea can be extended for AR rare pathogenic missense variants. Here, random clusters may support the identification of critical molecular regions^33,34^. For *VPS13B* no clustering of neither loss-of-function nor missense variants could be recognized so far^8^. At the Eighth International Chorea-Acanthocytosis Symposium it was agreed that characterization of rare human missense variants in VPS13 family members, which result in disease but do not cause loss of the specific VPS13 protein, is an important research topic^35^.

**Figure 1:**
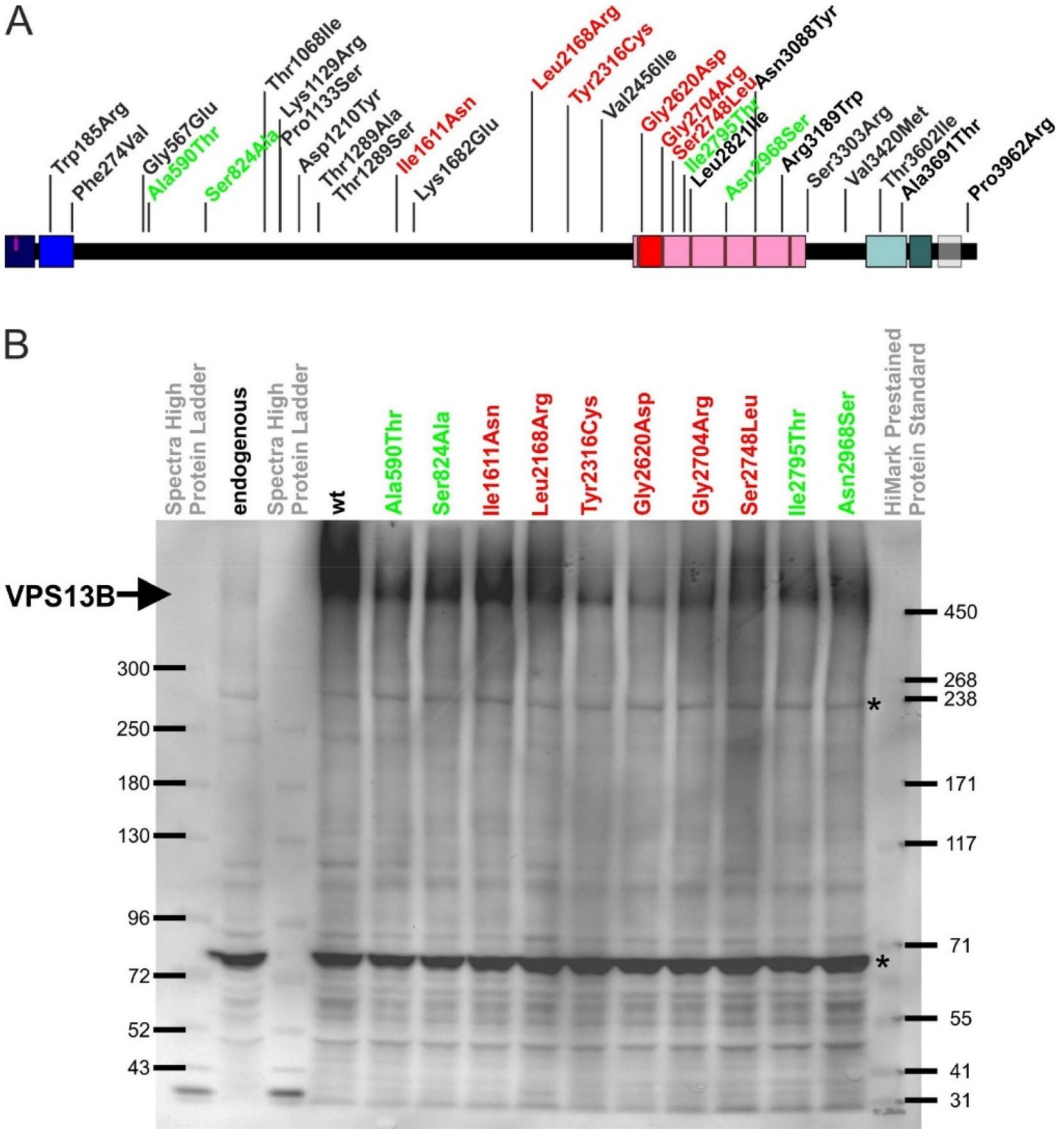
Protein stability of overexpressed wildtype and mutant VPS13B proteins. (**A**) Schematic representation of the VPS13B protein and localization of disease-associated missense variants. Potential functional or homology domains are indicated as follows: Dark blue: a Chorein/VPS13 region at the very N-terminus (aa 3–102, pfam12624), Blue: a second VPS13-N-terminal region (aa 139–280, pfam16908), Red: a Vps13-adaptor binding (VAB) domain (formerly known as classical DUF1162 or SHR-binding domain, aa 2603–2702), Pink: an extended VAB domain (aa 2715–3363, pfam06650)^43^, Mint: a VPS13-C-terminal region (aa 3543–3709, pfam16909), and Dark mint: an autophagy-related protein C terminal domain (aa 3713–3816, pfam09333). Color-coded VPS13B missense variants were in vitro analyzed for their Golgi complex association. Missense variants with Golgi association are green while missense variants which fail to associate at the Golgi complex are red. (**B**) HeLa cells were transfected with pcDNA3.1_VPS13B constructs. 24 h post-transfection cells were collected in RIPA lysis buffer, total protein concentration per lane was adjusted and lysates were processed for SDS-PAGE/Western blot analysis by VPS13B. Arrow indicates VPS13B at 450 kDa. *Indicates previously described false positive signals of the VPS13B (442) antibody^6^.

The mammalian VPS13 protein family consists of four homologues to yeast Vps13p–VPS13A (CHAC), VPS13B (COH1), VPS13C, and VPS13D (Supplementary Fig. S1)^36^. They can be classified as pioneer proteins, since their cell biological as well as molecular function cannot be concluded from sequence homologies. They have in common their giant size (over 300 kDa) and N- and C-terminal VPS13 homology domains^6,36^. To date, genetic alterations in all four *VPS13* genes have been described as causative for various human neurological disease syndromes. Variants in *VPS13A* cause neurodegenerative Chorea-Acanthocytosis^37,38^. Genetic defects in *VPS13B* lead to the neurodevelopmental Cohen syndrome and were also linked to autism, intellectual disability, retinal disease, primary immunodeficiency disease, and short stature^1,2,13–15,19,29,31^. Mutations in *VPS13C* are a rare cause of neurodegenerative early-onset Parkinson's disease^39,40^. And childhood onset of movement disorders is due to variants in *VPS13D*^41,42^. By sequence homologies searches, domain identification databases SMART (http://smart.embl.de/smart/) and pfam (http://pfam.xfam.org/), and literature comparison, we summarize a Chorein/VPS13 region at the very N-terminus (aa 3–102, pfam12624), a second VPS13-N-terminal region (aa 139–280, pfam 16908), a Vps13-adaptor binding (VAB) domain (formerly known as classical DUF1162 or SHR-binding domain, aa 2603–2702), an extended VAB domain (aa 2715–3363, pfam06650)^43^, a VPS13-C-terminal region (aa 3543–3709, pfam16909), and an autophagy-related protein C terminal domain (aa 3713–3816, pfam09333) in common with all other VPS13 proteins (Fig. 1A). In contrast to VPS13A, VPS13C, and VPS13D, VPS13B lacks further VPS13-N-terminal mid repeating regions (Supplementary Fig. S2). VPS13B contains in the 6 VAB repeat regions 4 out of 6 highly conserved VPS13B asparagines (manually curated in consistence with^44^). However, we were not able to predict the PH-domain and the APT1 domain at the VPS13B C-terminus using HHpred (https://toolkit.tuebingen.mpg.de/tools/hhpred)^45^ or Phyre2 (http://www.sbg.bio.ic.ac.uk/phyre2/)^46^, which has been studied for yeast Vps13p and VPS13A^47,48^. However, it is highly likely that these domains are conserved in all 4 mammalian VPS13 proteins (Supplementary Fig. S2).

All *VPS13*-associated diseases follow an AR inheritance mode, further emphasizing a loss-of-function disease mechanism. Here, we aim to determine the cell biological consequences of the hitherto known *VPS13B* missense variants and will explore their pathogenic potential.

## Materials and methods

### Study design

We searched the literature using PubMed and HCMG thoroughly to summarize all published disease-associated *VPS13B* missense variants. Clinical indication and published phenotypical signs were summarized (Supplementary Table T1). We included all mentioned *VPS13B* missense variants regardless of their final interpretation by the respective authors. All *VPS13B* missense variants were subjected to a systematically in silico analysis. Moreover, we searched the gnomAD database for allele frequencies of these missense variants. 10 out of 29 missense variants were cloned as *VPS13B* full length mutants to investigate their impact on Golgi complex function in vitro. Finally, we reclassified their pathogenicity according to the American Collage of Medical Genetics (ACMG) standards^49^.

### Variant pathogenicity prediction algorithms and ACMG classification

To assess the pathogenicity of SNPs in silico, a series of established and publicly accessible prediction algorithms were used: Grantham score^50^, PROVEAN^51^, PolyPhen-2^52^, MutationTaster^53^, SIFT^54^, MutPred^55^, and SNPs&GO^56^. Allele frequency was determined using the gnomAD database^57^. Variant classification according to ACMG criteria is a scoring system of precisely defined terms to estimate the impact of a genetic variant on a certain disease or beneficial health state^3,58^. The following information and ACMG terms were implement for classification of genetic *VPS13B* missense variants: (i) variant frequency in control populations (PM2), (ii) computational in silico prediction of the variant impact (PP3), (iii) functional analysis of the variant (PS3), (iv) segregation of the variant within the family (PP1 supporting), and (v) information about the specificity of the phenotype (PP4). The applied terms are summarized for each variant and result in rating a certain variant as pathogenic (P), likely pathogenic (LP), or variant of unknown significance (VUS).

### Reagents and antibodies

All reagents were obtained from Sigma-Aldrich, Roth or Merck unless otherwise stated. The following commercial antibodies were used: Mouse anti-GM130 (BD Transductions Laboratories, 610822, final conc. 2 ng/ml). Rabbit anti-VPS13B (442) was described earlier^6^. Secondary antibodies for Western blotting were anti-rabbit IgG-HRP and anti-mouse IgG-HRP (both DAKO GmbH) and for immunofluorescence donkey anti-mouse-Cy3, anti-rabbit-Cy3, and anti-rabbit-Cy2 (all Dianova GmbH). 6-Diamidino-2-phenylindole (DAPI) (Invitrogen) was applied for nuclear DNA-staining.

### Cloning of VPS13B mutant expression vectors by site-directed mutagenesis

Genetic variants were assembled from the literature. All missense variants were annotated according to the *VPS13B* (ENST00000357162.6 (*VPS13B*-202) (Ensemble)/NM_152564 (RefSeq)) transcript, including exon 28b in exchange for exon 28 with ubiquitous expression, 14,019 bp transcript length, and encoding 3997 amino acids (aa). For transient expression, full-length human *VPS13B* constructs containing the *VPS13B* missense variants (pcDNA3.1_VPS13B-mut) were cloned by site-directed mutagenesis PCR^59^. Briefly, full-length wild-type human construct pcDNA3.1_VPS13B-wt^6^ was amplified using primer pairs containing the corresponding missense variants in *VPS13B* by Phusion polymerase (NEB Inc.) according to manufacturer’s instructions. PCR-products were subsequently treated with endonuclease DpnI (NEB Inc.) to eliminate the methylated pcDNA3.1_VPS13B-wt vectors and further processed for standard DNA ligation reaction. The integrity and correct mutagenesis of all cloned vectors was confirmed by Sanger sequencing.

### Cell culture and transfection

HeLa-cells (ATCC) were cultured in DMEM, 5% FCS, 1% glutamine at 37 °C, 5% CO_2_. Transient transfection of expression vectors was performed at 60–75% confluence using jetPEI (Polyplus transfection) according to manufacturer’s instructions. Cell harvesting for further analysis was performed 24–48 h after transfection. RNA interference was performed using *VPS13B*-specific siRNA (Ambion); sequences are available on request. The mixture of siRNA, INTERFERin (Polyplus transfection), and OptiMEM (ThermoFisher) was transfected to the cells twice in 12 h intervals according to manufacturer’s protocol. The cells were harvested 72 h after the first transfection.

### Quantitative PCR

Isolation of mRNA from HeLa cell-lysates was performed with Isol-RNA Lysis Reagent (5 Prime) according to manufacturer’s manual, followed by cDNA synthesis using the M-LV reverse transcriptase (Promega). qPCR analysis was conducted on Fast Real-Time PCR 7500 System (Applied Biosystems) according to the 2^−ΔΔCT^ method^60^ using the HOT FIREPol EvaGreen HRM (Solis BioDyne) and *VPS13B*-specific primers (sequences available on request). All samples were determined in triplets and normalized against GAPDH expression. Statistical analysis was done by SDS2.1 software (Applied Biosystems).

### Immunofluorescence

For visualization of intracellular protein expression, cells were cultivated on coverslips and if required transfected (see above). Cells on coverslips were fixed using 3% (w/v) paraformaldehyde in PBS at 4 °C and permeabilized in 0,5% (v/v) Triton X-100,1% (w/v) BSA in PBS. Primary antibodies in 1% (w/v) BSA in PBS were incubated for at least 8 h at 4 °C. After rinsing with PBS, secondary antibodies in 1% (w/v) BSA in PBS were incubated for 2 h at 4 °C. Subsequently, coverslips were attached to slides using Fluoromout G (SoutherBiotech). Visualization occurred on the LSM 5 EXCITER confocal microscope and images were analyzed using the ImageJ-software and according macros.

### SDS-PAGE/western blotting

All samples were boiled in 1 × Laemmli buffer and subsequently separated by gel electrophoresis in NuPAGE 3–8% Tris–acetate SDS polyacrylamide gradient gels (Invitrogen) together with Spectra Multicolour High Range Protein Ladder (Thermo Fisher) and HiMark Prestained Protein Standard (life technologies) as range indicators. Separated proteins were transferred to nitrocellulose membranes. After blocking with 5% block milk, membranes were incubated with corresponding primary and HRP-bound secondary antibodies in 5% block milk. Visualization was performed using ECL reaction (Western Blotting Luminol, Santa Cruz Biotechnology) on X-Ray Film (Fuji Medical Super RX).

### ImageJ and statistical analysis

Golgi enrichment was assessed using ImageJ. Two different regions of interest (ROI) were defined for each cell separately: one was drawn around the outline of the cell to detect whole cell total immunofluorescence of VPS13B and the other was drawn around GM130-positive Golgi structure to detect Golgi-associated immunofluorescence of VPS13B. The percentage of Golgi-associated VPS13B fluorescence per total VPS13B cell fluorescence intensity was calculated. The graphical representation as well as the statistical analysis (Dunnett’s multiple comparision test) was carried out using GraphPad Prism Software.

## Results

To gain further insight into functional VPS13B regions, we summarized literature-published disease-associated VPS13B missense variants, validated their clinical context, predicted the molecular genetic impact, investigated selected variants experimentally, and finally interpreted their disease relevance according to ACMG (Supplementary Fig. S1).

### Systematic in silico analyses of published rare disease-associated missense variants in *VPS13B*

First, we performed a profound literature search on missense variants in *VPS13B* in cases of Cohen syndrome, autism, intellectual disability, retinal disease, primary immunodeficiency disease, and short stature using Pubmed (https://www.ncbi.nlm.nih.gov/pubmed/) and HGMD (http://www.hgmd.cf.ac.uk/ac/index.php). Overall, 29 *VPS13B* disease-associated missense variants were identified^1,2,8,13–23,25–31,61,62^ (Table 1). The positions of these missense variants within the VPS13B protein are shown in Fig. 1A. We referenced all *VPS13B* variants to the evolutionary conserved and ubiquitously expressed transcript [NM_152564.4, ENST00000357162] encoding a protein of 3997 aa. However, the annotation to the longest transcript [NM_017890.4, ENST00000358544], which is specifically expressed in the brain and comprises 4022 aa^8^, is also given in Supplementary Table T1.

**Table 1:** Summary of results from in vitro characterization of the hitherto cloned VPS13B missense variations and update on ACMG classification.

Protein change (NP_689777.3)^a^	dbSNP	ClinVar (clinical significance)	Gnomad (minor allele frequency)	Gnomad (homozygous)	Described phenotype	Golgi localization	Golgi rescue	Applicable ACMG pathogenicity criteria	ACMG classification
Wildtype	–	–	No data	No data	–	Yes	Yes		
Trp185Arg	–	No data	No data	No data	Cohen syndrome	n.d	n.d		
Phe274Val	–	No data	0.0004442	0	Autism	n.d	n.d		
Gly567Glu	rs141046414	Uncertain significance	0.0008347	**2**	Undiagnosed genetic condition	n.d	n.d		
**Ala590Thr**	**rs140601319**	**Uncertain significance**	0.0001592	**1**	**Cohen syndrome**	**Yes**	**Yes**	**None**	**VUS**
**Ser824Ala**	**rs149866274**	**Uncertain significance**	0.01056	35	**Autism**	**Yes**	**Yes**	**None**	**VUS**
Thr1068Ile	rs61753722	Benign/likely benign	0.004610	5	Epilepsy	n.d	n.d		
Lys1129Arg	rs61759485	Conflicting interpretations	0.000003981	0	Polymorphism	n.d	n.d		
Pro1133Ser	rs781781537	Likely pathogenic	No data	No data	Undiagnosed genetic condition	n.d	n.d		
Asp1210Tyr	–	No data	0.00009212	0	Retinal disease, CS	n.d	n.d		
Thr1289Ala	rs752808333	Uncertain significance	0.0003788	1	Primary immunodeficiency disease	n.d	n.d		
Thr1289Ser	rs145569846	Conflicting interpretations	no data	**No data**	Cohen syndrome	n.d	n.d		
**Ile1611Asn**	**–**	**No data**	0.000003540	0	**Cohen syndrome**	**No**	**No**	**PM2, PP3, PS3, PP1sup, PP4**	**LP**
Lys1682Glu	rs965365158	No data	No data	**No data**	Primary immunodeficiency disease	n.d	n.d		
**Leu2168Arg**	**–**	**Pathogenic**	0.000007075	**0**	**Cohen syndrome**	**No**	**No**	**PM2, PP3, PS3, PP4**	**LP**
**Tyr2316Cys**	**rs386834104**	**Likely pathogenic**	0.0001768	0	**Cohen syndrome**	**No**	**No**	**PM2, PP3, PS3, PP4**	**LP**
Val2456Ile	rs201963516	Conflicting interpretations	no data	**No data**	Intellectual disability	n.d	n.d		
**Gly2620Asp**^b^	**–**	**Conflicting interpretations**	0.000007975	**0**	**Cohen syndrome**	**No**	**No**	**PM2, PP3, PS3, PP1sup, PP4**	**LP**
**Gly2704Arg**	**rs767536787**	**No data**	0.000003985	**0**	**Cohen syndrome/Autism**	**No**	**No**	**PM2, PP3, PS3**	**LP**
**Ser2748Leu**	**rs180177370**	**No data**	No data	**No data**	**Cohen syndrome**	**No**	**No**	**PM2, PP3, PS3, PP1sup, PP4**	**LP**
**Ile2795Thr**	**–**	**Pathogenic**	No data	No data	**Cohen syndrome**	**Yes**	**Yes**	PM2, PP4*	**VUS**
Leu2821Ile		No data	0.003096	**1**	n/p	n.d	n.d		
**Asn2968Ser**	**rs28940272**	**Conflicting interpretations**	no data	No data	**Cohen syndrome**	**Yes**	**Yes**	**PP3, PP1sup**	**VUS**
Asn3088Tyr	–	No data	0.003778	4	Short stature	n.d	n.d		
Arg3198Trp	rs149842139	Benign/likely benign	no data	No data	Autism	n.d	n.d		
Ser3303Arg	–	No data	0.000202	0	Autism/ Cohen syndrome	n.d	n.d		
Val3445Met	rs191174682	Benign/likely benign	0.000007960	0	Cohen syndrome	n.d	n.d		
Thr3602Ile	–	No data	0.002538	5	Autism/Cohen syndrome	n.d	n.d		
Ala3691Thr	rs142476821	Conflicting interpretations	No data	No data	Autism/ Cohen syndrome	n.d	n.d		
Pro3962Arg	–	Uncertain significance	No data		Autism	n.d	n.d		

Missense variants in bold were cloned and tested for Golgi localization/rescue and further reclassified according to ACMG guidelines.

^a^Conservation of the affected amino acids is given in Supplementary Table T2.

^b^Missense variant in combination with consanguineous homozygous loss-of-function variant; *n.d.* not determined.

In order to assess the clinical impact of these 29 *VPS13B* missense variants, the genotype and phenotypical characteristics of these cases were summarized (Supplementary Information and Supplementary table T1). The phenotype of each case was critically reviewed by the literature data to assess the probability of a Cohen syndrome diagnosis (Supplementary Information and Supplementary Table T1). The probability of a Cohen syndrome diagnosis, was estimated by using a score counting the main diagnostic criteria: facial dysmorphism, microcephaly, intellectual disability, myopia/retinopathy, neutropenia, and other Cohen syndrome-supportive features (Supplementary Table T1). Although not obligatory, especially neutropenia and/or retinopathy in combination with intellectual disability and microcephaly are clearly pointing toward a Cohen syndrome diagnosis. Accordingly, 19 out of 35 cases demonstrate with a Cohen syndrome consenting phenotype. Several patients from one family were summarized as one case and at least 2 out of 6 criteria (criterium needs to be =/> 50%) have to be extracted from the literature. In 13 out of 35 cases the score for Cohen syndrome was not determinable due to the lack of informative phenotype data. Cases from this group are mostly from different large-scale next generation sequencing (NGS)-screens focusing on one specific clinical sign such as autism, short stature, retinal disease, primary immunodeficiency, and undiagnosed genetic conditions independent of further syndromic features. In three out of 35 cases the Cohen syndrome phenotype score for was < 50%. In this group are: 1 patient with autism, 1 patient with epilepsy and a compound heterozygous so far not confirmed splice site variant c.8016+7G>C, and 1 patient with phenotypically suspected Cohen syndrome but only one heterozygous missense variant. However, two further cases are healthy carriers of one heterozygous missense variant.

All 29 missense variants were evaluated using in silico analyses to predict potential pathogenicity at protein level. These approaches followed three types: (1) sequence and evolutionary conservation-based method, (2) protein sequence and structure-based methods, and (3) supervised machine learning methods. Moreover, the minor allele frequency (MAF) was extracted from GnomAD browser^63^. The accumulated in silico results suggest 12 *VPS13B* missense variants with very low MAF and high disease probability by in silico prediction (Trp185Arg, Asp1210Tyr, Ile1611Asn, Lys1682Glu, Leu2168Arg, Tyr2316Cys, Gly2620Asp, Gly2704Arg, Ser2748Leu, Asn3088Tyr, Ser3303Arg, and Thr3602Ile) as potentially disease-causing (Table 1, Supplementary Table T2). In addition, 10 *VPS13B* variants were identified with low disease-causing potential by high MAF and negative in silico prediction (Gly567Glu, Ala590Thr, Ser824Ala, Thr1068Ile, Lys1129Arg, Thr1289Ser, Asn2968Ser, Arg3198Trp, Val3420Met, and Ala3691Thr).

### Protein levels of full-length VPS13B remained unaffected by missense variants

The selection on *VPS13B*-associated missense variants for in vitro studies was based on a clear Cohen syndrome/autism association, family-based validation of the genetic finding, and low MAF (Fig. 1A, Table 1). Eight VPS13B missense variants were selected for further in vitro analysis (encoding Ala590Thr, Ile1611Asn, Leu2168Arg, Tyr2316Cys, Gly2620Asp, Ser2748Leu, Ile2795Thr, and Asn2968Ser). These were chosen because prior to the introduction of NGS, larger intragenic deletions and duplications, which are also a common cause of Cohen syndrome, were often overlooked on the second allele^64^. The selection further comprises two missense variants (encoding Ser824Ala and Gly2704Arg), which were identified in a NGS-based screening on autism^31^. To assess the functional impact of these disease-associated missense variants, we subcloned the hitherto selected 10 missense variants into the pcDNA3.1_VPS13B full-length-wild-type (VPS13B-wt) expression vector^6^. To test, whether all VPS13B-mut vectors express normal protein levels, we performed transient overexpression experiments in HeLa cells. The VPS13B-wt as well as all VPS13B-mut proteins are detectable at 450 kDa with similar expression levels suggesting that none of the missense variants cause protein instability (Fig. 1B). None of the mutant VPS13B proteins showed abnormalities with regard to protein degradation or altered general expression.

### Golgi fragmentation is rescued by co-overexpression of wildtype-VPS13B

**Figure 2:**
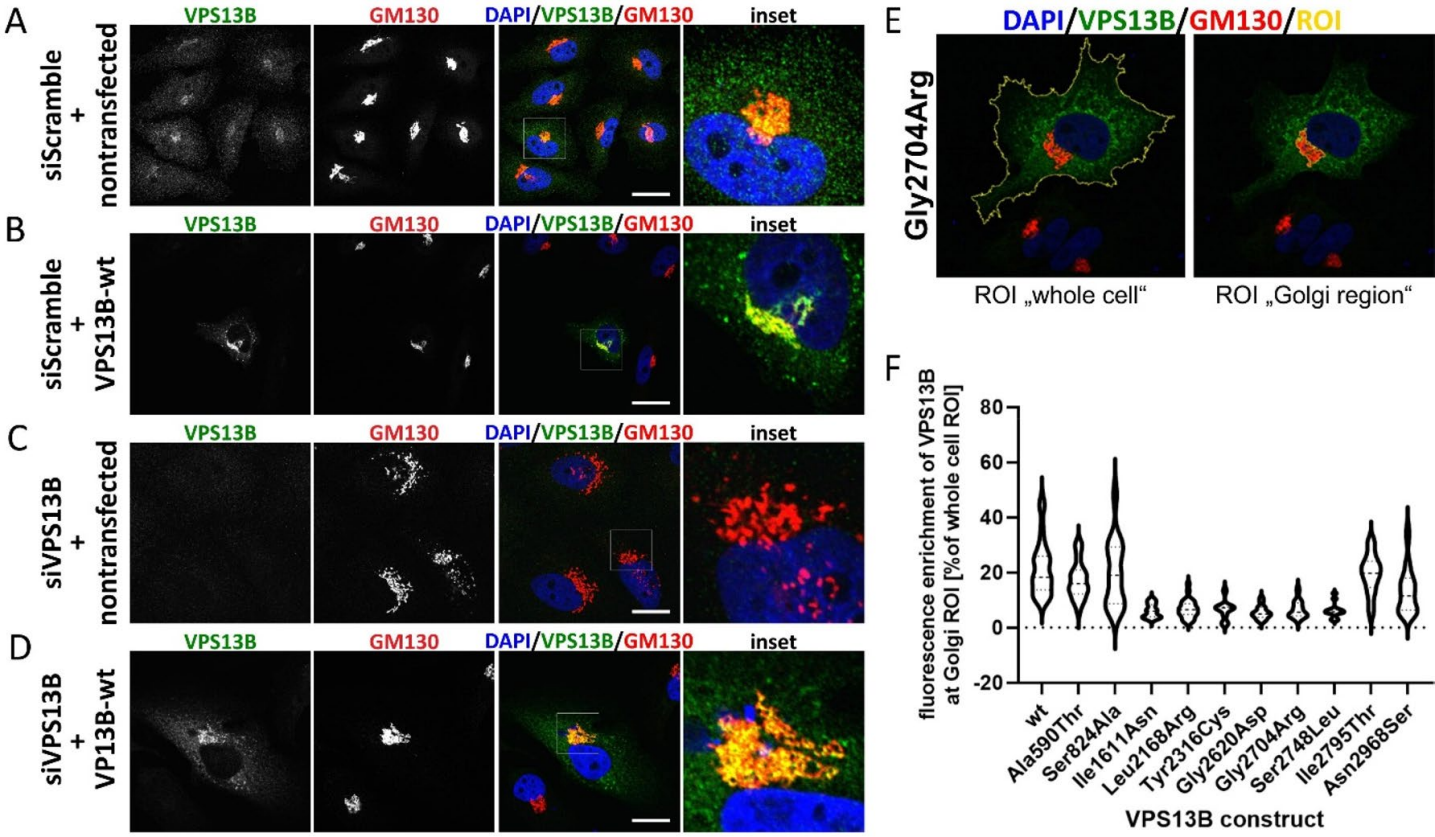
Golgi enrichment is affected by VPS13B missense variants. (**A**–**D**) Golgi fragmentation is rescued by wild type VPS13B overexpression. HeLa cells were transfected with either control (scrambled siRNA) (**A**,**B**) or a *VPS13B*-specific RNAi (siRNA #25721, ambion) (**C**,**D**). 48 h post RNAi-transfection HeLa cells were processed for overexpression and either nontransfected (**A**,**C**) or transfected with the pcDNA3.1_VPS13B-wt construct (**B**,**D**). 24 h later, all cells were processed for immunostaining of VPS13B (green) and GM130 (red). Imaging occurred with confocal microscopy. Nuclei were stained using DAPI, scale bars 10 µm. (**E**,**F**) Analysis of subcellular localization of cloned VPS13B missense variants. ImageJ analysis was used to evaluate Golgi localization of VPS13B missense variants. Two ROI (yellow) were defined for each cell: “whole cell” ROI was drawn at the outline of the cell to detect total immuofluorescence levels; “Golgi region” was defined by the counterstaining of GM130 (red). Fluorescence intensity of VPS13B (green) in each ROI was measured (**E**). Percentage of VPS13B enrichment at the Golgi was finally calculated for each missense variant using GraphPad prism. We repeated each condition at least 3 times and analyzed each time 3 recordings (with at least 1 transfected cell). Please refer to Supplementary table T3 for statistical information (**F**).

Immunohistochemical staining and confocal microscopy of transiently transfected HeLa cells revealed that endogenous as well as overexpressed VPS13B-wt co-localized with the Golgi complex marker (GM130) (Fig. 2A,B). This is consistent with previous observations^6^. To exclude possible off-target effects of the hitherto used *VPS13B*-specific siRNA, we established a rescue approach. This includes after *VPS13B* knockdown subsequent transient transfection with a VPS13B-wt expression vector (pcDNA3.1_VPS13B-wt). Effective knockdown of *VPS13B* was confirmed by qPCR (Supplementary Fig. S3) and absence of an appropriate VPS13B protein signal at GM130 positive structures (Fig. 2C). The resulting VPS13B depletion led to severe Golgi fragmentation (Fig. 2C). The Golgi fragmentation and co-localization with GM130 was restored upon by overexpression of VPS13B-wt (Fig. 2D).

### Six VPS13B missense variants do not localize to the Golgi complex

VPS13B is critical for structural integrity of the Golgi complex. VPS13B-deficiency has been shown to fragment Golgi cisternae in fibroblasts derived from Cohen syndrome patients and in *VPS13B*-siRNA knockdown experiments^6^. To assess the subcellular localization of the VPS13B-mut proteins, expression vectors with the corresponding VPS13B missense variant were transfected into Hela cells and the subcellular localization was assessed with immunohistochemical staining, confocal imaging and, fluorescence intensity measurement. Four VPS13B-mut proteins—Ala590Thr, Ser824Ala, Ile2795Thr, and Asn2968Ser—localized to the Golgi complex and showed a similar staining pattern as VPS13B-wt. However, six missense variants—Ile1611Asn, Leu2168Arg, Tyr2316Cys, Gly2620Asp, Gly2704Arg, and Ser2748Leu—did not co-localize with GM130 but demonstrated with a diffuse cytoplasmic staining pattern (Fig. 2E,F, and Supplementary Fig. S4). The significance was calculated using the Dunnett’s multiple comparison test GraphPad prism and details of the analysis are provided in the supplement (Supplementary Table T3).

### Six VPS13B missense variants do not rescue siRNA-induced Golgi fragmentation

**Figure 3:**
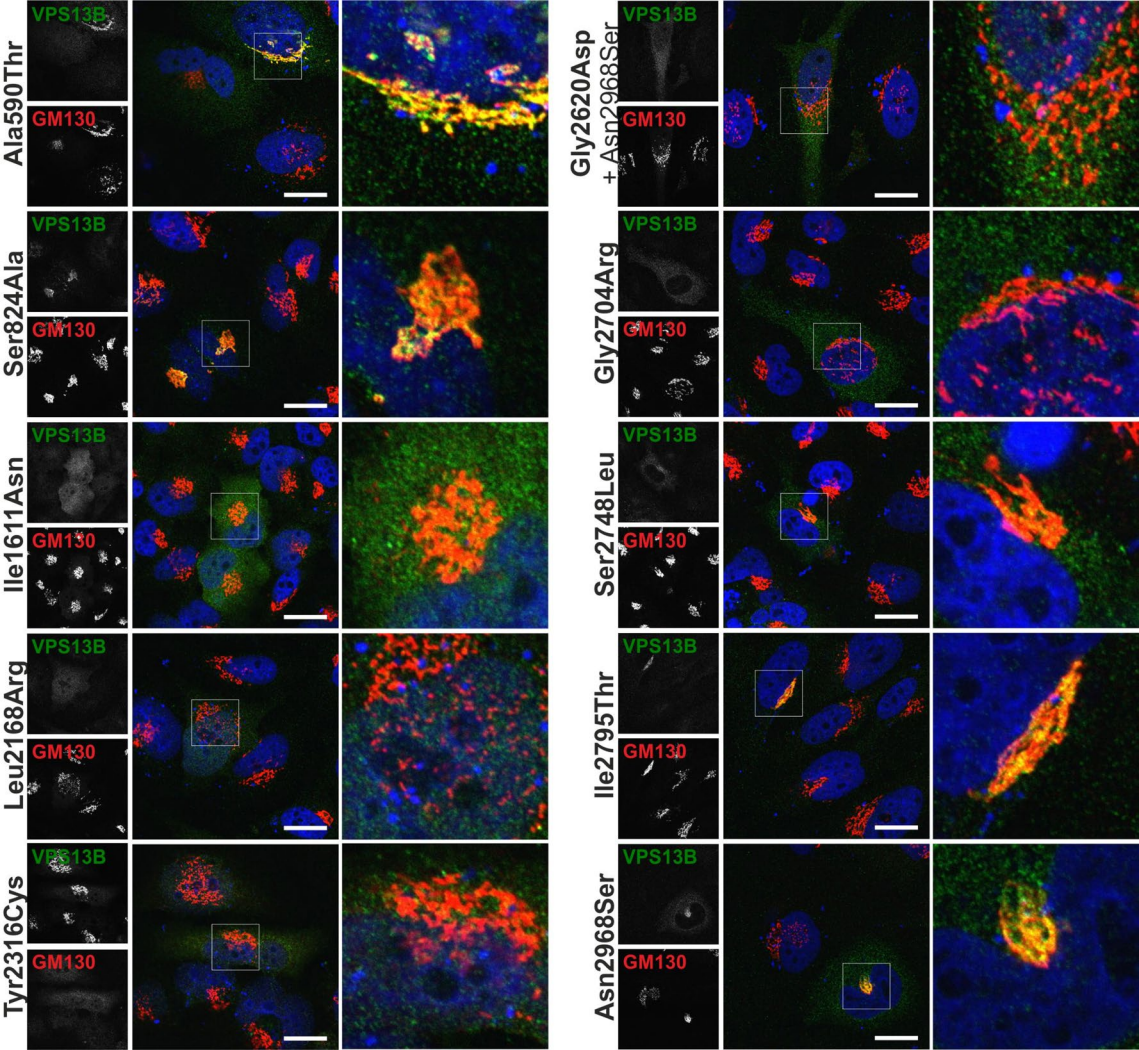
Rescue experiment of Golgi fragmentation by co-overexpression of VPS13B missense variants. HeLa cells were transfected with a *VPS13B*-specific RNAi (siRNA #25721, ambion). 48 h post RNAi-transfection HeLa cells were transfected with the respective pcDNA3.1_VPS13B-mut constructs. 24 h later, cells were processed for immunostaining of VPS13B (green) and GM130 (red). Imaging occurred with confocal microscopy. Nuclei were stained using DAPI, scale bars 10 µm.

To further validate the functional overlap of VPS13B-mut compared to VPS13B-wt, we performed a rescue experiment. HeLa cells were treated with a *VPS13B*-specific siRNA inducing fragmentation of the Golgi complex and subsequently transfected with VPS13B-mut vectors. Golgi integrity was assessed with immunohistochemical staining as well as confocal imaging. VPS13B-wt recovered the compact Golgi structure comparable to untreated controls (Fig. 2D). An equivalent effect was observed in four missense variants—Ala590Thr, Ser824Ala, Ile2795Thr, and Asn2968Ser (Fig. 3). In contrast, the other six VPS13B-mut vectors—Ile1611Asn, Leu2168Arg, Tyr2316Cys, Gly2620Asp, Gly2704Arg, and Ser2748Leu—did not restore the compact Golgi morphology and resulted in a diffuse cytoplasmic VPS13B staining.

### Six VPS13B missense variants are classified as likely pathogenic according to ACMG guidelines

For ACMG variant classification we applied input data from multiple levels: MAF from gnomAD population data (PM2), delirious impact of a variant according to computational evidence (PP3), experimental/functional data (PS3), segregation information (PP1), and gene specific phenotype (PP4) (Supplementary Table T2)^3^.

Six out of the 10 experimentally tested *VPS13B* missense variants were classified as LP variants (Table 1). The remaining 4 experimentally analyzed variants were classified as VUS. The strongest applied terms for LP classification were very low MAF from gnomAD population data (PM2) and negative functional impact of VPS13B-mut on Golgi organization from in vitro analysis (PS3). It is important to note that 5 *VPS13B* LP variants were detected in patients with a clear diagnosis of Cohen syndrome and in 1 patient with autism. Moreover, the 6 *VPS13B* LP variants were genetically detected in homozygous state in consanguine families or in compound heterozygous state with a *VPS13B* truncating variant.

## Discussion

Missense variants are part of the genetic variability within the healthy population, but some usually very rare variants are responsible for genetically inherited diseases. Classifying of rare missense variants with disease states is difficult but provides association with precise genetic defects. Here we assessed the relevance of rare *VPS13B* missense variants for the development of Cohen syndrome. An improved understanding of *VPS13B* missense variants in Cohen syndrome and the affected cellular processes are essential to provide mechanistic explanations and will help to elucidate the disease pathomechanisms.

The members of the VPS13 protein family are considered as pioneering proteins exposing little structural or sequence homologies to other proteins. Recent studies using TAP-tag purified yeast Vps13p and single-particle electron microscopy identified an elongated rod-like structure (~ 4 × 20 nm) with a loop (internal diameter ~ 6 nm) at the one and a hook at the opposite end of yeast Vps13p^70^. Further studies on a N-terminal fragment from *Cheatomium thermophilum* VPS13 by single particle cryo-EM indicate that the N-terminus is composed of beta-strands forming a long channel-like structure facilitating lipid transport between membranes^65^.

Here, six disease-associated VPS13B missense variants were found to disrupt the Golgi complex localization of the VSP13B protein. Moreover, three of these VPS13B missense variants localize in or rather in close proximity with the VAB domain (Gly2620Arg, Gly2704Arg, Ser2748Leu). Although, the function of the VAB domain is not fully understood, recent data suggest that the VAB domain mediates interaction to phosphatidylinositol 3-phosphate (PI3P) in yeast Vps13^66^. Moreover, the VPS13A/CHAC missense variant Ile2771Arg attenuates interaction with PI3P suggesting a role of the VAB domain for direct membrane tethering. Mapping of three VPS13B missense variants to the VAB domain is remarkable as it suggests a similar role of this region for VPS13B. However, limited experimental protein structure information and in silico data prediction hinders assessment of the other three VPS13B missense variants Ile1611Asn, Leu2168Arg, and Tyr2316Cys for overall protein function.

Ongoing utilization of missense variants for functional exploration of VPS13B requires analysis of their disease-causing potential. This analysis involves genetic information, clinical data, in silico predictions, and experimental evidence. Here, we show that functional analysis of disease-associated *VPS13B* missense variants at the Golgi complex critically contributes to variant classification. In this regard, we demonstrate that 6 out of 10 experimentally analyzed *VPS13B* missense variants can be classified as LP according to ACMG. Strongest rating was contributed by the experimental data (PS3) and the low MAF in gnomAD (PM2) stressing the importance of functional read out approaches and population frequency databases for genetic variant interpretation. Together with this, the other four experimentally tested variants are classified as VUS so far. All these variants showed higher MAF values, weak in silico prediction strength, and/or negative experimental outcome suggesting them as variants without or uncertain functional impact. Further in vitro studies are clearly required to confirm or refute their pathogenicity.

Currently, genetic and phenotype databases foster the implementation of ACMG classified genetic variants. ClinVar is a free accessible archive of clinically relevant variants, which has received an increasing number of submission due to routine genetic testing with NGS^67^. When searching for *VPS13B*, ClinVar shows more than 1000 listed variants. Out of those, 877 *VPS13B* missense variants are listed, most of them as VUS (788), five as LP, and three as P. Eight out of the 29 hitherto summarized missense variants are not listed within ClinVar (Supplementary Table T2). Two out of 29 *VPS13B* missense variants are listed as P (Ile1611Asn, Leu2168Arg) and one variant as LP (Pro1133Ser) (Table 1, https://www.ncbi.nlm.nih.gov/clinvar). Here we confirmed the variants Ile1611Asn and Leu2168Arg as P. Moreover, 4 missense variants (Tyr2316Cys, Gly2620Asp, Gly2704Arg, and Ser2748Leu), which are listed in ClinVar with conflicting interpretations or as VUS, can be classified as LP due to our in vitro study. The provided classification for the *VPS13B* missense variant Pro1133Ser as LP in ClinVar should be recategorized as VUS according to the ACMG guidelines^18^. All *VPS13B* missense variants of this study with a functional impact on Golgi integrity were identified in patients with a definite diagnosis of Cohen syndrome. No genotype–phenotype correlations can currently be performed for Cohen syndrome or *VPS13B*-associated autism. Further research will identify missense variants affecting other VPS13B protein functions and may describe different VPS13B-associated phenotypes in more detail.In conclusion, our study emphasizes that careful and conservative ACMG evaluation as well as consequent database curation is required to avoid mis-, over-, or underinterpretation of genetic *VPS13B* missense variants. This impacts a broad spectrum of diseases and genes in medical genetics^58,68,69^. Consequently, careful investigation and classification of VPS13B missense variants will help to solicit genetic counseling, medical management, and psychosocial guidance of patients as well as their families.

Altogether, our study provides first in vitro evidence that rare *VPS13B* missense variants play a critical role for the pathogenesis of Cohen syndrome. These missense variants compromise, at least partially, the VPS13B protein function. Utilization of further disease-associated missense variants and additional in vitro studies will fundamentally support functional assessment of the VPS13 protein family members and foster classification of VPS13B missense variants, respectively.

## Supplementary Information

Supplementary Information.
