# Supplementary material for: Disease relevance of rare VPS13B missense variants for neurodevelopmental Cohen syndrome
Source: Sci Rep. 2022 Jun 11;12:9686. doi: 10.1038/s41598-022-13717-w (PMC9188546; doi:10.1038/s41598-022-13717-w)
Supplement: Supplementary file 1 — Supplementary Information. [file 41598_2022_13717_MOESM1_ESM.pdf]

SUPPLEMENT

Disease relevance of rare *VPS13B* missense variants for neurodevelopmental Cohen syndrome

Malte Zorn[1*], Jirko Kühnisch[2*], Sebastian Bachmann[1], Wenke Seifert[3]

From the Charité - Universitätsmedizin Berlin, corporate member of Freie Universität Berlin, Humboldt-Universität zu Berlin, and Berlin Institute of Health, Institute of Vegetative Anatomy, Berlin, Germany[1], Charité - Universitätsmedizin Berlin, corporate member of Freie Universität Berlin, Humboldt-Universität zu Berlin, and Berlin Institute of Health, Experimental and the Clinical Research Center (ECRC), a joint cooperation between the Charité Medical Faculty and the Max-Delbrück-Center for Molecular Medicine, Berlin, Germany[2], Charité - Universitätsmedizin Berlin, corporate member of Freie Universität Berlin, Humboldt-Universität zu Berlin, and Berlin Institute of Health, Institute of Cell Biology and Neurobiology, Berlin, Germany[3], [*]contributed equally

Correspondence

Wenke Seifert, Institute of Cell Biology and Neurobiology, Charité - Universitätsmedizin Berlin, Charitéplatz 1, 10117 Berlin, Germany, E-mail: wenke.seifert@charite.de

Summary on literature reported disease-associated *VPS13B* missense variants

Six out of 29 disease-associated *VPS13B* missense variants were reported in compound heterozygosity or homozygosity with two further most likely truncating variants on both alleles (Lys1129Arg, Asp1210Tyr, Thr1289Ser, Ile2795Thr, Ser3303Arg, and Val3420Met. Thus, those rare missense variants can be suspected to be more likely benign [33, 34].

Five out of 29 disease-associated *VPS13B* missense variants were reported in compound heterozygosity with one most likely truncating variant on the second allele (Trp185Arg, Thr1068Ile, Leu2168, Tyr2316Cys, and Gly2704Arg).

Seven out of 29 disease-associated *VPS13B* missense variants were reported in different cases as homozygous variants (Ser824Ala, Ile1611Asn, Val2456Ile, Gly2620Asp, Ser2748, Asn2968Ser, and Arg3198Trp). Asn2968Ser was originally identified in a consanguine family in homozygosity [20]. However, the same mutation was more recently in compound heterozygosity with another missense variant Leu2821Ile [29]. For the missense variant Arg3198Trp in autism conflicting genetic data have been reported [15]. This variant is inherited by both male children in homozygosity from an unaffected homozygous father and an unaffected heterozygous mother in one family, and by another male patient in heterozygosity from an unaffected heterozygous father and an unaffected heterozygous mother in another family. For the latter family no further *VPS13B*-associated variation was identified.

Five plus one out of 29 disease-associated *VPS13B* missense variants were reported in compound heterozygosity with another missense variant (Gly567Glu, Pro1133Ser, Leu2821Ile, Thr3602Ile, and Ala3691Thr; plus Asn2968Ser).

Six out of 29 disease-associated *VPS13B* missense variants were reported in heterozygous inheritance where a second variant is still unknown (Phe274Val, Ala590Thr, Thr1289Ala, Lys1682Glu, Asn3088Tyr, and Pro3962Arg). Here, the three missense variants Phe274Val, Thr1289Ala, and Leu1682Glu) have been reported in targeted NGS for autism or primary immunodeficiency disease screens in occurrence with other heterozygous variants in further disease-causing genes [4, 21].The results from *in vitro* characterization of the hitherto cloned *VPS13B* missense variations and their update on ACMG classification are summarized in table 1.

Details on literature reported disease-associated *VPS13B* missense variants

The missense variant Trp185Arg was identified in one male with intellectual disability, microcephaly and joint hypermobility; combining typical features of Cohen syndrome [36]. At the age of 3 years clinical assessment did not reveal any ophthalmic or blood problems. The missense variant was detected as compound heterozygous with the fatal missense variant Cys733*.

The missense variant Phe274Val was identified in a male patient and was found in heterozygosity, lacking further variants in *VPS13B* [21]. The patient belonged to a study in which target gene enrichment of autism-associated genes occurred. However, a second heterozygous missense variant was identified in RAI1. The authors state that this missense variation is most unlikely disease causing.

The missense variant Gly567Glu was identified in compound heterozygosity with another Pro1133Ser missense variant during diagnostic family-based exome sequencing [11]. All patients were found to have a genetic etiology but lack a genetic diagnosis. Functional follow-up evidence of the hitherto identified missense variants remains elusive.

The missense variant Ala590Thr was identified as heterozygous in one female offspring from nonconsanguineous Italian parents [18]. A compound heterozygous second variant in *VPS13B* is missing so far. Age of clinical assessment was 24 years. The Cohen syndrome phenotype is incomplete without documented microcephaly, retinopathy and myopia. However, moderate intellectual disability and neutropenia indicate a Cohen syndrome.

The missense variant Ser824Ala was identified in a male patient with incomplete Cohen syndrome phenotype [37]. The analysis was performed in a screening of genetic causes for autism and was found

in homozygous state. The authors speculate an incomplete, hypomorph Cohen syndrome due to reduced protein availability.

The missense variant Thr1068Ile was identified in compound heterozygosity with a mild intragenic c.8016+7G > C, which has not been tested for its effect on the splicing event [36]. The major phenotype of the patient were repeated seizures and very mild intellectual disability.

The missense variant Lys1129Arg was identified in two siblings with Cohen syndrome [33]. However, both affected children carrier two compound heterozygous LoF variations in *VPS13B*, which segregate within the family and were therefore considered as solely responsible to cause Cohen syndrome. In a more recent study this missense variant reappeared in a genetic approach on patients with retinal disease [35]. The respective patient has a compound heterozygous pathogenic variant in *VPS13B* (c.10232delC) and was clinically assessed to have Cohen syndrome.

The missense variant Pro1133Ser was identified in compound heterozygosity with the above described Gly567Glu missense variant [11].

The heterozygous missense variant Asp1210Tyr was identified in a whole-genome sequencing approach on patients with retinal disease [3]. The respective patient presents with two other compound heterozygous pathogenic variants in *VPS13B* (Ser864* and Met2124Valfs*44), probably solely causative for the inherited retinal disease.

The heterozygous missense variant Thr1289Ala occurred in a targeted disease genes approach for primary immunodeficiency [4]. A second variant in *VPS13B* could not be identified; however, further heterozygous variants were identified in the respective patient in other primary immunodeficiency-associated disease genes: *SLC37A4, SCNN1G*, and *CXCR4*.

The missense variant Thr1289Ser in the second child of a nonconsanguineous Caucasian African couple was identified in combination with a paternal inherited 3 bp insertion (c. 11752_11753insATG) and a maternal inherited 315 kb deletion spanning from exon 4 of the *OSR2* gene to exon 17 of the *VPS13B* gene (chr8: 100015029…100347846del) [30]. Phenotypic assessment occurred at the age of 2 years with Cohen syndrome-like facial dysmorphism but without typical ophthalmological and/or hematological Cohen syndrome-associated findings.

The homozygous missense variant Ile16111Asn was recognized in Moroccan twins from consanguineous parents [9]. Both twins show typical Cohen syndrome-like features including intellectual disability, microcephaly, facial dysmorphism, and slender extremities.

The heterozygous missense variant Lys1682Glu occurred in a targeted disease genes approach for primary immunodeficiency [4]. A second variant in *VPS13B* could not be identified; however, further heterozygous variants were identified in the respective patient in other primary immunodeficiency-associated disease genes: *ADA, FERMT3*, and *CD79B*.

The compound heterozygous missense variant Leu2168Arg was found in combination with a 2 bp deletion (c.3348_3349delCT) in a finish patient with typical a finish phenotype of Cohen syndrome [19].

The missense variant Tyr2316Cys was identified as compound-heterozygous together with a nonsense variation (c.11240C>T) [13]. Clinical assessment of the 4-yearold female showed typical facial dysmorphism, intellectual disability and postnatal microcephaly. However, ophthalmological and hematological findings were unremarkable which is most likely due to the younger age of the patient at clinical assessment.

The homozygous missense variant Val2456Ile was identified in an exome sequencing approach in persons with severe intellectual disability [7]. The male patient was clinically assessed at the age of 4 years and presents with intellectual disability and microcephaly but normal motor development. MRI of the brain was normal, and at the age of around one year he lost his previously accomplished communication skills.

The missense variant Gly2620Asp was identified in two consanguine families from the Oman. All patients share a typical facial dysmorphism, intellectual disability, myopia and microcephaly in agreement with Cohen syndrome [13, 26]. Moreover, the oldest child (10 years at clinical assessment) showed initial ophthalmological abnormalities indicative of retinopathy and her MRT screening showed a comparable enlarged corpus callosum [26].

The missense variant Gly2704Arg was identified in a screening of genetic causes for autism [37]. However, the clinical reevaluation of the patient showed typical Cohen syndrome-like features. Moreover, the missense variant was identified to be compound heterozygous with a nonsense variation in *VPS13B* (c.8110G>A).

The missense variant Ser2748Leu was identified in two consanguineous families [34]. All three patients (two female, one male) showed typical Cohen syndrome-like facial appearance and intellectual disability. Two patients presented also with postnatal microcephaly. Due to their younger age (8, 5 and 2.5 years) the exclusion of later onset of ophthalmological problems could not be excluded.

The missense variant Ile2795Thr was recognized in 8 patients from an Amish community in US, Ohio [10]. However, all 8 patients carry an additional homozygous insertion (c.9183_9184insT) in *VPS13B*, which is most likely solely causative for the typical Cohen syndrome phenotype in all those patients. In another clinical study on bilateral angle closure glaucoma the missense variant Ile2795Thr was identified in heterozygosity in the described patient with Cohen syndrome. The patient has Amish background and was further genetically tested carrying the homozygous c.9258_9259insT [24].

The missense variant Leu2821Ile was detected in a whole-exome sequencing screen across clinical indicators with focus on family-based analysis and classified as very likely pathogenic [29]. However, from the supported material, one cannot clearly extract status of heterozygosity, inheritance or clinical presentation of the belonging patient.

The missense variant Asn2968Ser was identified in homozygous occurrence in Belgian twins [20]. Both patients presented with a typical Cohen syndrome including intellectual disability, microcephaly, neutropenia, retinopathy and myopia. However, the authors themselves speculated that this missense variant could represent a rare nonpathogenic change. This missense variant reoccurred in in a whole-exome sequencing screen across clinical indicators with focus on family-based analysis [29]. However, from the supported material, one cannot clearly extract status of heterozygosity, inheritance or clinical presentation of the belonging patient. In a large-scale genomic WES project on 267 genomes representing the healthy Spanish population the missense variant Asn2968Ser occurred one time on one allele [8].

The heterozygous missense variant Asn3088Tyr was identified in a genetic research study on short stature by target/whole exome sequencing [14]. A second variant in *VPS13B* was not identified. In addition to the short stature, the female patient was born with an atrial septal defect and mitral regurgitation and presents with intellectual disability as well as microcephaly. However, according to the ACMG guidelines, the authors categorized this missense variant as variance of uncertain significance.

The missense variant Arg3198Trp was identified in a sequenced-based study on identification of rare causal variants in Cohen syndrome and autism [15]. The variant appeared in 2 families, each with 2 affected siblings. The total allele count was 5 in affected individuals versus one in unaffected individuals. Reevaluation occurred by Sanger sequencing. However, the transmission showed also homozygosity in the unaffected father of one family.

The missense variant Val3445Met (c.10333G>A) was identified in heterozygosity with a homozygous Tyr413* (c.1239T>G) by whole-exome sequencing and was confirmed by Sanger sequencing. [23]. The patient presented with Cohen syndrome-agreeable features including normal head circumference at birth and postnatal microcephaly, intellectual disability and a typical facial appearance.

The missense variant Thr3602Ile (c.10805C>T) was identified on same allele in very close proximity to a deletion c.10808_10825delCGAGGCAGCTTGTGCACG [9]. Thus, it is more likely that both alterations occurred at the same DNA changing event. However, they were found to be compound heterozygous with nonsense variation (Arg692*). All patients with *VPS13B* variations had intellectual disability, a Cohen syndrome-typical facial dysmorphism, microcephaly and slender extremities with narrow hand/feet. Moreover, 11/12 patients had neutropenia.

The missense variants Ser3303Arg and Ala3691Thr were identified as compound heterozygous in one patient with an autisms-like phenotype [37]. However, reevaluation showed Cohen-syndrome-like facial dysmorphism but normocephalic brain development.

The heterozygous missense variant Pro3962Arg was identified in a male patient, lacking a second *VPS13B*-associated variant [21]. The patient belonged to a study in which target gene enrichment of autism-associated genes occurred. The authors state that this missense variation is most unlikely disease causing.

Genotype and phenotypical characteristics of these previously reported patients are summarized in supplementary table T1.

In silico analyses of disease-associated _VPS13B_ missense variants

To study missense variants in *VPS13B*, we started with a literature search on PubMed (https://www.ncbi.nlm.nih.gov/pubmed/). 29 missense variants were retrieved as associated with Cohen syndrome, autism, intellectual disability, retinal disease, primary immunodeficiency disease, or short stature [3, 4, 7, 9-11, 13-15, 17, 19-21, 26, 29, 30, 33-37]. However, this search warrants no completeness. We reevaluated all 29 missense variants using several established pathogenicity prediction algorithms for *in silico* analysis, which can be broadly subdivided into three types: (1) sequence and evolutionary conservation-based method, (2) protein sequence and structure-based methods and (3) supervised machine learning methods.

The Grantham substitution matrix is one of the earliest and simplest methods and provides the prediction of effects in substitutions between amino acids (aa) based in their chemical properties, including polarity and molecular volume. The Grantham score subdivides the substitutions by their increasing chemical dissimilarity in 4 different classes: conservative (0-50), moderately conservative (51-100), moderately radical (101-150), or radical (≥151) [12]. For the hitherto analyzed 29 missense variants, the Grantham substitution matrix identified three variants (Asp1210Tyr, Ile1611Asn and Tyr2316Cys) as radical, eight as moderately radical (Trp185Arg, Leu2168Arg, Gly2704Arg, Ser2748Leu, Asn3088Tyr, Arg3198Trp, Ser3303Arg, and Pro3962Arg), 12 as moderately conservative (Gly567Glu, Ala590Thr, Ser824Ala, Thr1068Ile Pro1133Ser, Thr1289Ala, Thr1289Ser, Lys1682Glu, Gly2620Asp, Ile2795Thr, Thr3602Ile, and Ala3691Thr), and six as conservative (Phe274Val, Lys1129Arg, Val2456Ile, Leu2821Ile, Asn2968Ser, and Val3420Met). However, the Grantham score exclusively assesses the chemical dissimilarity of the exchanged substitution without regard to protein conservation, structure, domains and/or motifs. PROVEAN (protein variation effect analyzer, http://provean.jcvi.org) and SIFT (http://sift.jcvi.org/, sorting intolerant from tolerant) algorithms are comparable analysis tools for filtering sequence variants based on sequence homology. PROVEAN includes the quality of sequence alignment of the flanking neighborhood sequences [5, 6]. Thus, the prediction accuracy is based on the supporting set of homologous sequences which is influenced by the choice of underlying protein databases. PROVEAN discriminates its score thresholds between deleterious (lower than default -2.500) and neutral (higher than -2.500). To increase specificity (>90%) a lower score threshold (-4.100) can be used, while to increase sensitivity (>90%) a higher threshold can be used (-1.300). For the hitherto analyzed 29 missense variants, the PROVEAN algorithm identified 13 variants as deleterious (Trp185Arg, Asp1210Tyr, Ile1611Asn, Leu2168Arg, Tyr2316Cys, Gly2620Asp, Gly2704Arg, Ser2748Leu, Asn2968Ser, Asn3088Tyr, Ser3303Arg, Thr3602Ile, and Ala3691Thr) and 16 variants as neutral. SIFT is a multi-step algorithm, which basically scans each position for evolutionary conservation and calculates all probabilities [22, 27]. SIFT uses a multiple sequence alignment (MSA) strategy to assess

the probability of a deleterious effect. A highly conserved residue is generally assumed to be intolerant to substitutions while low conserved residues tolerate most substitutions. SIFT discriminates in damaging scores (≤0.05) and tolerated (≥0.05) scores. For the hitherto analyzed 29 missense variants, the SIFT algorithm identified 27 variants as damaging and only two variants as tolerated (Val2456Ile and Ala3691Thr). The Polyphen-2 (http://genetics.bwh.harvard.edu/pph2/, polymorphism phenotyping) provides an algorithm for filtering sequence variants based on structural homology [1]. Polyphen-2 discriminates in three different categories – probably damaging (<0.96), possibly damaging (<0.2-<0.96) and benign (>0.2). For the hitherto analyzed 29 missense variants, the Polyphen-2 algorithm identified 14 variants as probably damaging (Trp185Arg, Lys1129Arg, Asp1210Tyr, Thr1289Ala, Thr1289Ser, Ile1611Asn, Tyr2316Cys, Gly2620Asp, Gly2704Arg, Ser2748Leu, Asn2968Ser, Asn3088Tyr, Arg3198Trp, and Ala3691Thr), nine variants as possibly damaging (Phe274Val, Ala590Thr, Thr1068Ile, Lys1682Glu, Leu2168Arg, Ile2795Thr, Leu2821Ile, Ser3303Arg, and Thr3602Ile) and six variants as tolerated (Gly567Glu, Ser824Ala, Pro1133Ser, Val2456Ile, Val3420Met, and Pro3962Arg). Further MutPred2 (http://mutpred.mutdb.org/, random forest method), MutationTaster2 (http://mutationtaster.org/, Bayes classifier) and SNP&GOAnalyzer (http://snps.biofold.org/snps-and-go/snps-and-go.html) are machine learning-based algorithms, which combine genetic and molecular data to predict the probability of pathogenicity of amino acid substitutions. MutPred2 discriminates at a cut-off of 0.5 between pathogenic and non-pathogenic predictions [28]. Here, 17 out of 29 missense variants have been classified as pathogenic variants (Trp185Arg, Phe274Val, Thr1068Ile, Pro1133Ser, Asp1210Tyr, Ile1611Asn, Lys1682Glu, Leu2168Arg, Tyr2316Cys, Gly2620Asp, Gly2704Arg, Ser2748Leu, Asn2968Ser, Asn3088Tyr, Ser3303Arg, Thr3602Ile, and Ala3691Thr). With respect for missense variants, MutationTaster2 integrates information spanning evolutionary conservation, different biochemical databases and established analyses tools [31, 32]. The output distinguishes disease causing from polymorphism. Regarding the VPS13B-missense variants, except for 4 missense variants (Ser824Ala, Val2456Ile, Val3420Met, and Arg3198Trp) all other variants were predicted to be disease causing. SNPs&GO, a support vector machine (SVM) method, combines information on sequence (multiple sequence alignment, MSA), alleged protein function (Gene Ontology, GO) and Panther Evolutionary Analysis of Coding SNPs results [2, 25]. The SNPs&GO is similar to the PhD-SNP method. Both methods differentiate between neutral and disease. For the SNPs&GO algorithm 5 out of 29 have been classified as disease (Trp185Arg, Asp1210Tyr, Tyr2316Cys, Gly2704Arg, and Val3420Met). According to PhD-SNP, 12 out of 29 missense variants have been classified as neutral (Phe274Val, Gly567Glu, Ala590Thr, Ser824Ala, Thr1068Ile, Lys1129Arg, Thr1289Ala, Thr1289Ser, Val2456Ile, Leu2821Ile, Val3420Met, and Ala3691Thr). Further analytical tools implementing higher order protein structures (e.g. nsSNPAnalyzer, ERIS, Clustal, ConSurf, I-Tasser, SuperPose, ClusPro) could not be carried out due to the lack of further information on the protein structure of VPS13B. Allele frequency was extracted from GnomAD browser [16]. All prediction results are summarized in supplementary table T2.

Supplementary figure S1: **Study design**. The study design involved *in silico* and *in vitro* analysis to predict and investigate the impact of *VPS13B* missense variants (P - pathogenic, LP - likely pathogenic, VUS - variant of unknown significance, LB - likely benign, B - benign).

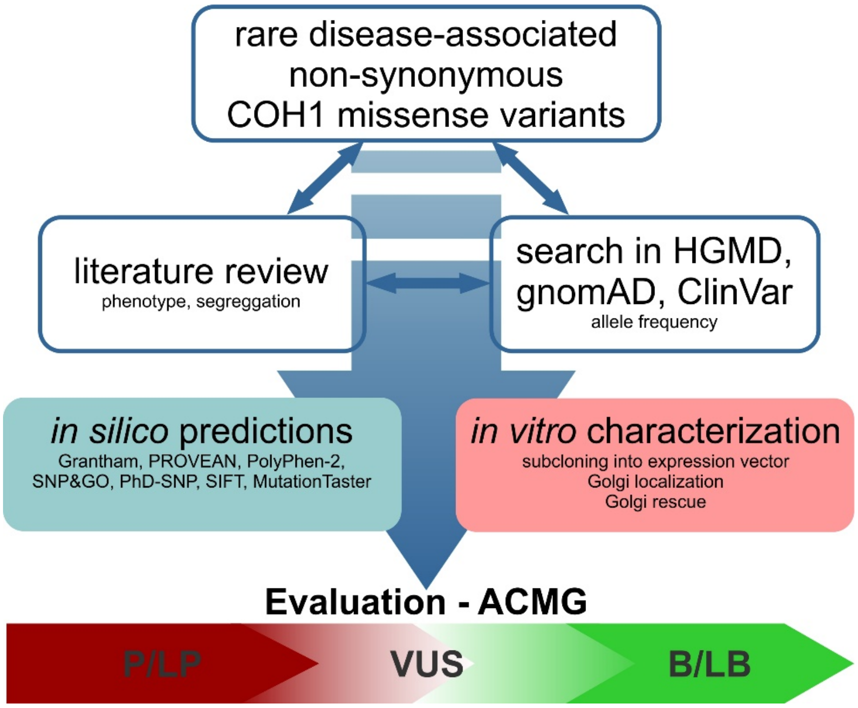

Supplementary Figure S2: **VPS13 family.** The figure summarizes the protein length and domain organization of yeast Vps13p and human VPS13 family members VPS13A (CHAC), VPS13B (COH1), VPS13C, and VPS13D.

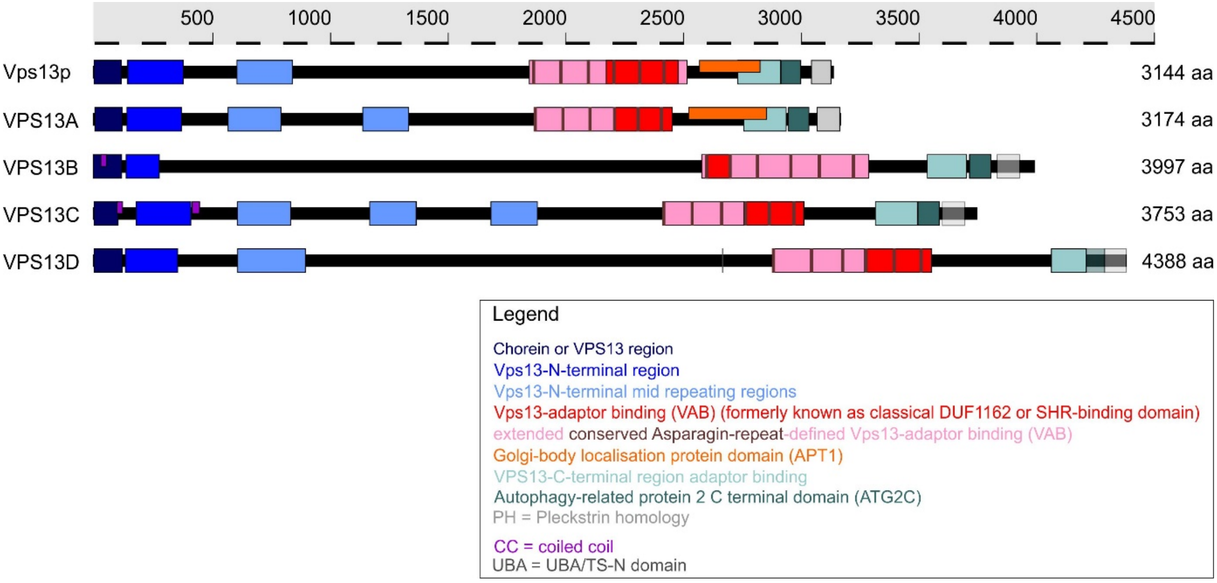

Supplementary Figure S3: Expression analysis with qPCR assessing relative *VPS13B* transcript levels during siRNA treatment and transient overexpression in HeLa cells.

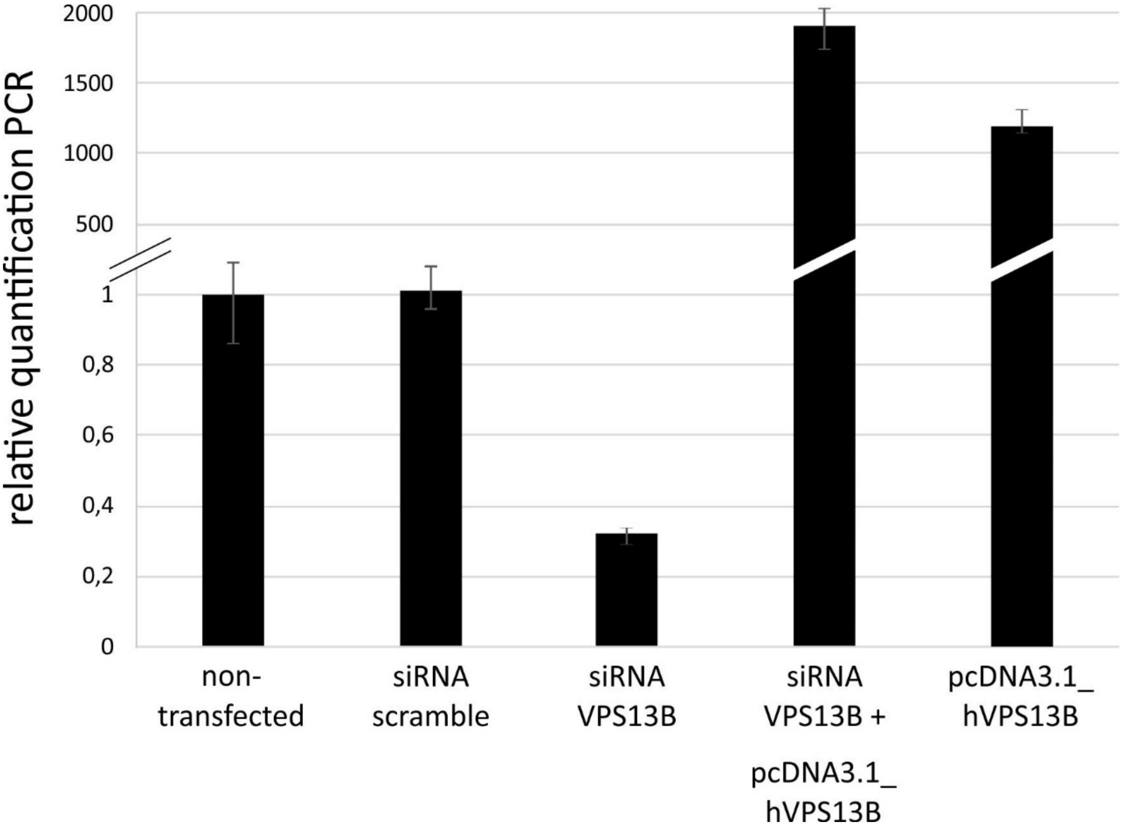

Supplementary Figure S4: **Localization of mutant VPS13B in HeLa cells.** HeLa cells were transfected with the respective pcDNA3.1_VPS13B-mut constructs. 24h later, cells were processed for immunostaining of VPS13B (green) and GM130 (red). Imaging occurred with confocal microscopy. Nuclei were stained using DAPI (blue), scale bars 10 µm.

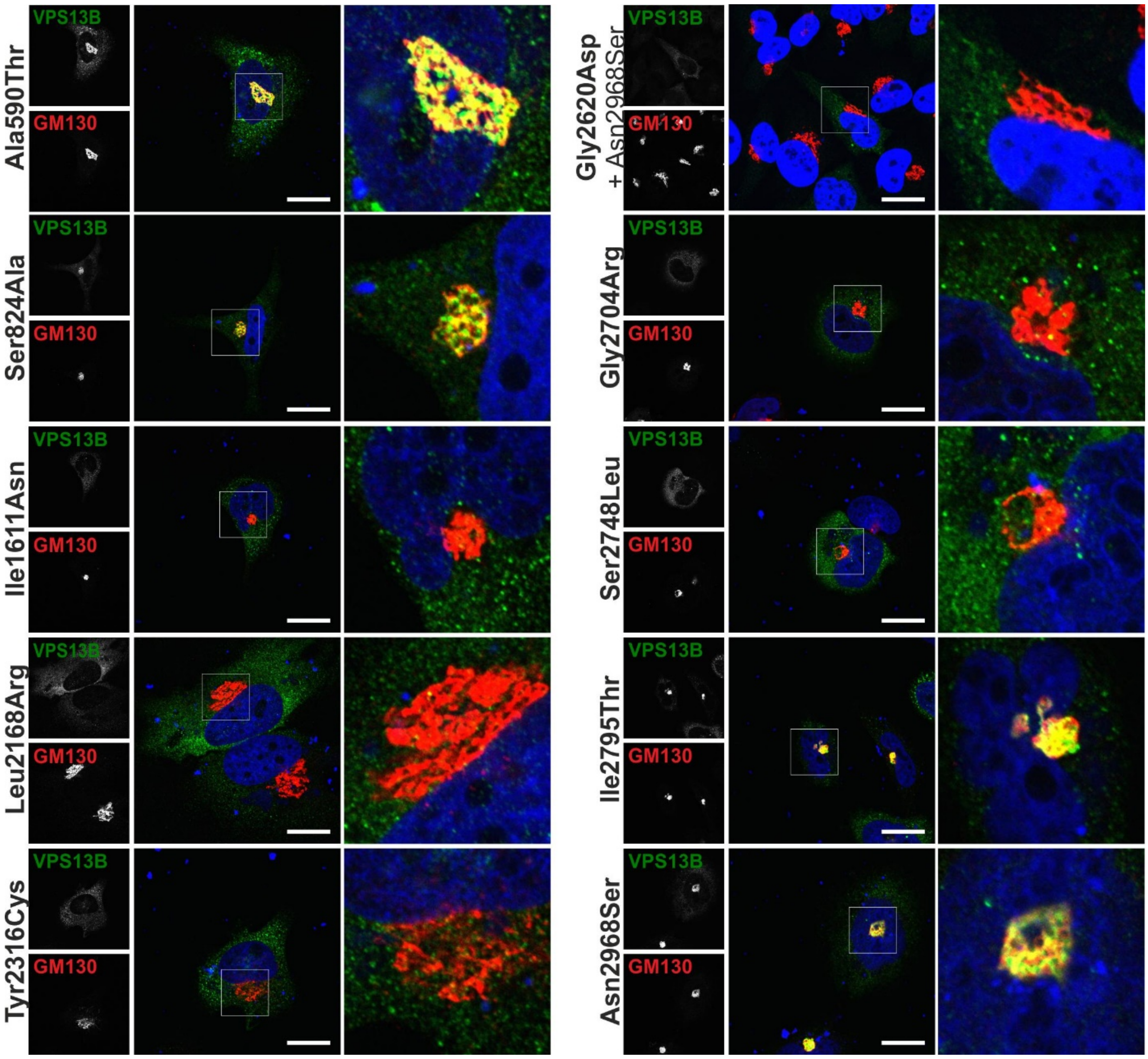

Supplementary Table T1: Summary of clinical symptoms of patients with missense variants in *VPS13B*.

Protein change (NP_689777.3)	cDNA Change (NM_152564.4)	Protein change (NP_060360.3)	cDNA Change (NM_017890.4)	Exon	Inheritance	Sex	Age at diagnosis (a)	I. Facial Dys-morphism	II. Micro-cephaly	III. Intellectual Disability	IV. Myopia/ Retino-pathy	V. Neutro-penia	VI. Other CS features	Initial Diagnosis	Score for CS	Detection method	Reference
Trp185Arg	c.553T>C	Trp185Arg	c.553T>C	5	compound heterozygous with Cys733*	M	3	+	++	++	-	-	++[1]	CS	3.5/6	WGS (NextSeq500, illumina)	Yang et al., 2018
Phe274Val	c.820T>G	Phe274Val	c.820T>G	7	heterozygous; second variant unknown; another missense variant het in RAI1	M	11	n/p	n/p	++	n/p	n/p	-[4]	Autism	1/2	target gene enrichment (PGM, Thermo Fisher Scientific versus MiSeq, illumina)	Koshimizu et al. 2013
Gly567Glu	c.1700G>A	Gly567Glu	c.1700G>A	13	compound heterozygous with Pro1133Ser	n/p	n/p	n/p	n/p	n/p	n/p	n/p	n/p	undiagnosed genetic condition	n.d.	diagnostic WES (SureSelect Target Enrichment System, Agilent Technologies or SeqCap EZ VCRome 2.0, Roche NimbleGen)	Farwell et al., 2015
**Ala590Thr**	c.1768G>A	Ala590Thr	c.1768G>A	13	heterozygous; second variant unknown	F	24	- (yes/no)	-	++	-	+	+[2]	CS	2/6	Sanger sequencing, DHPLC, TaqMan	Katzaki et al. 2007
**Ser824Ala**	c.2470T>G	Ser824Ala	c.2470T>G	17	homozygous, consanguine	M?	n/p	+	n/p	n/p	n/p	n/p	+[1]	Autism	1/2	WGS (HiSeq, illumina), confirmed by Sanger sequencing	Yu et al. 2013
Thr1068Ile	c.3203C>T	Thr1068Ile	c.23278C>T	22	compound heterozygous with c.8016+7G>C	M	5	-	-	+	-	-	-[4]	Epilepsy	0.5/6	WGS (NextSeq500, illumina)	Yang et al., 2018
Lys1129Arg	c.3386A>G	Lys1129Arg	c.3386A>G	23	compound heterozygous with c.10232delC	n/p	n/p	n/p	n/p	n/p	++	n/p	n/p	Retinal disease/CS	n.d.	target specific sequencing, SNP calling and chromosome analysis (Sanger sequencing; EP1 system, Fluidigm ; T-ARMS PCR, chromosomal microarray)	Stone et al., 2017
					compound heterozygous with 2 other CS causing variants (Gln2229Hisfs*10/ Trp3649*)	F,F	8,5	++	++	++	++	++	+	CS	5.5/6	Sanger sequencing	Seifert et al. 2009
Pro1133Ser	c.3397C>T	Pro1133Ser	c.3397C>T	23	compound heterozygous with Gly567Glu	n/p	n/p	n/p	n/p	n/p	n/p	n/p	n/p	undiagnosed genetic condition	n.d.	diagnostic WES (SureSelect Target Enrichment System, Agilent Technologies or SeqCap EZ VCRome 2.0, Roche NimbleGen)	Farwell et al., 2015
Asp1210Tyr	c.3628G>T	Asp1210Tyr	c.3628G>T	24	compound heterozygous with 2 other CS causing variants (Ser864*/ Met2124Valfs*44)	F	n/p	n/p	n/p	n/p	++	n/p	n/p	Retinal disease, CS	1/1	WES and WGS	Carss et al., 2017
Thr1289Ala	c.3865A>G	Thr1289Ala	c.3865A>G	27	heterozygous, second variant unknown; other het variants in SLC37A4, SCNN1G, CXCR4	M	2	n/p	n/p	n/p	n/p	++	-[6]	Primary immunodefi-ciency disease	1/1	targeted HTS (Agilent SureDesign with NextSeq500, illlumina)	Chi et al., 2018
Thr1289Ser	c.3866C>G	Thr1289Ser	c.3866C>G	27	compound heterozygous with 2 other CS causing variants (ex01-17del/ Asp3942_Gly3943insAsp)	F	2	++	-	++	-	-	n/p	CS	2.5/5	Genome-wide screen for CNVs (SNP array 6.0, affymetrix))	Rivera-Brugués et al. 2011
**Ile1611Asn**	c.4832T>A	Ile1636Asn	c.4907T>A	31	homozygous, consanguine	n/p;n/p	n/p; n/p	++	++	++	n/p	n/p	+[3]	CS	3.5/4	Sanger sequencing	El Chehadeh et al. 2010
Lys1682Glu	c.5044A>G	Lys1707Glu	c.5119A>G	32	Heterozygous; second variant unknown; other	M	1	n/p	n/p	n/p	n/p	++	-[5]	Primary immunodefi-ciency disease	1/1	targeted HTS (Agilent SureDesign with NextSeq500, illlumina)	Chi et al., 2018

					het variants in ADA, FERMT3, CD79B												
**Leu2168Arg**	c.6503T>G	Leu2193Arg	c.6578T>G	37	compound heterozygous with Cys1092fs*7	n/p	n/p	++	++	++	++	++	+[1]	CS	5.5/6	Sanger sequencing	Kolehmainen et al. 2003
**Tyr2316Cys**	c.6947A>G	Tyr2341Cys	c.7022A>G	39	compound heterozygous with Gln3747*	F	4	++	++	++	+	-	++[1,2,3]	CS	4.5/6	Sanger sequencing	Hennies et al. 2004
Val2456Ile	c.7366G>A	Val2481Ile	c.7441G>A	41	homozygous	M	4	++	++	++	n/p	n/p	n/p[9]	Intellectual disability	3/3	diagnostic WES	de Ligt et al., 2012
**Gly2620Asp**	c.7859G>A	Gly2645Asp	c.7934G>A	43	homozygous, consanguine	F;M;M	7,5,2	++	++	++	++	n/p	++[1,2,3]	CS	5/5	Sanger sequencing	Hennies et al. 2004
**Gly2704Arg**	c.8110G>A	Gly2795Arg	c.8185G>A	45	compound heterozygous with Trp963*	M	n/p	++	n/p	n/p	n/p	n/p	n/p	Autism/CS	1/1	NGS (HiSeq, illumina), confirmed by Sanger sequencing	Yu et al. 2013
**Ser2748Leu**	c.8243C>T	Ser2773Leu	c.8318C>T	45	homozygous, consanguine	F,F<br>M	8,5<br>2	++<br>++	++<br>++	++<br>++	++<br>n/p	n/p<br>n/p	+[2]<br>-	CS	4.5/5<br>3/4	Sanger sequencing	Seifert et al.2006
**Ile2795Thr**	c.8384T>C	Ile2820Thr	c.8459T>C	46	homozygous, but consanguine amish people with second homozygous Leu3062fs*19<br>compound heterozygous amish with homozygous c.9258_9259insT	M,F,F,M,F<br>F,F,F<br>F	15,9,4,9,7<br>6,14,13<br>28	++<br>++<br>++	++<br>++<br>n/p	++<br>++<br>++	++<br>++<br>++	++<br>++<br>++	++<br>++<br>++	CS<br>CS<br>CS	6/6<br>6/6<br>5/5	Sanger sequencing<br>n/p	Falk et al.2004<br>Li et al 2018
Leu2821Ile	c.8461C>A	Leu2846Ile	c.8536C>A	47	compound heterozygous with Asn2968Ser	n/p[10]	n/p	n/p	n/p	n/p	n/p	n/p	n/p	n/p	n.d.	WES	Retterer et al., 2016
**Asn2968Ser**	c.8903A>G	Asn2993Ser	c.8978A>G	49	homozygous, consanguine<br>compound heterozygous with Leu2821Ile<br>heterozygous	n/p;n/p<br>n/p[10]<br>n/p	n/p;n/p<br>n/p<br>n/p	++<br>n/p<br>-	++<br>n/p<br>-	++<br>n/p<br>-	++<br>n/p<br>-	++<br>n/p<br>-	++<br>n/p<br>-	CS<br>n/p<br>healthy	6/6<br>n.d.<br>0/6	Sanger sequencing<br>WES<br>WES	Kolehmainen et al. 2004<br>Retterer et al., 2016<br>Dopazo et al. 2016
Asn3088Tyr	c.9262A>T	Asn3113Tyr	c.9337A>T	51	heterozygous, second variant is missing	F	1	-	++	++	n/p	n/p	+[7,8]	Short stature	2.5/3	target sequencing (ClearSeq Inheried Disease panel) and WES (SureSelect All Exon V5, Agilent with HiSeq4000, illumina)	Huang et al., 2018
Arg3198Trp	c.9592C>T	Arg3223Trp	c.9667C>T	52	4 patients:<br>1 homozygous<br>3 heterozygous cases with unknown second variant<br>1 control: heterozygous	n/p<br>n/p<br>n/p<br>n/p<br>n/p	n/p<br>n/p<br>n/p<br>n/p<br>n/p	n/p<br>n/p<br>n/p<br>n/p<br>n/p	n/p<br>n/p<br>n/p<br>n/p<br>n/p	n/p<br>n/p<br>n/p<br>n/p<br>n/p	n/p<br>n/p<br>n/p<br>n/p<br>n/p	n/p<br>n/p<br>n/p<br>n/p<br>n/p	n/p<br>n/p<br>n/p<br>n/p<br>n/p	Autism<br>Autism<br>Autism<br>Autism<br>healthy	n.d.<br>n.d.<br>n.d.<br>n.d.<br>n.d.	WES	Ionita-Laza et al., 2014
Ser3303Arg	c.9907A>C	Ser3328Arg	c.9907A>C	56	compound heterozygous with 2 other CS causing variants c.10808_10825delCGAGGCAGCTTGTGCACG/ Arg692*	n/p	n/p	++	++	++	n/p	n/p	+[3]	CS	3.5/4	Sanger sequencing	El Chehadeh et al. 2010
Val3420Met	c.10258G>A	Val3445Met	c.10333G>A	56	compound heterozygous with homozygous stop CS causing variant	F	1	++	++	++	n/p	n/p	+[3,11, 12]	CS	4/4	WES (Agilent SureSelect XT Human All Exon v5 kit) confirmed by Sanger sequencing	Lee et al. 2020
Thr3602Ile<br>Ala3691Thr	c.10805C>T<br>c.11071G>A	Thr3627Ile<br>Ala3716Thr	c.10880C>T<br>c.11146G>A	5?<br>58	compound heterozygous	M	n/p	+	-	n/p	n/p	n/p	+	Autism	1/3	NGS (HiSeq, illumina), confirmed by Sanger sequencing	Yu et al. 2013

Pro3962Arg	c.11885C>G	Pro3987Arg	c.11960C>G	62	heterozygous, second variant is unknown	M	12	n/p	n/p	++	n/p	n/p	+[2]	Autism	1.5/2	target gene enrichment (PGM, Thermo Fisher Scientific versus MiSeq, illumina)	Koshimizu et al.,2013

whole genome sequencing (WGS), whole exome sequencing (WES), ++ clear phenotype, + incomplete phenotype, - no phenotype, n/p not provided by literature; [1]joint hyperextensibility, [2]truncal obesity, [3]narrow hand/feet, [4]epilepsy, [5]recurrent respiratory tract infections, [6]recurrent cough and fever, [7]atrial septal defect; mitral regurgitation, [8]developmental delay of bilateral frontotemporal lobe gyrus, [9]normal motor development, lost communication skills at the age of 1 year, [10]abnormalities of the nervous system, [11] hearing loss, [12] cleft palate, micrognathia, glossoptosis

Supplementary Table T2: Computational predictions of pathogenicity and frequency (gnomAD, release August 2019) of the hitherto summarized missense variants in VPS13B.

Protein change (NP_689777.3)	Grantham Score	PROVEAN Score	Polyphen-2 (HumVar)	MutPred 2 Probability	SNPs&GO – PhD-SNP	SNPs&GO – SNPs&GO	SIFT Score	MutationTaster (Probability)	gnomAD Allele Count TOTAL	gnomAD Allele Number TOTAL	gnomAD Number of Homozygotes TOTAL	gnomAD Allele Frequency TOTAL	gnomAD Other variants at same position (Allele Number TOTAL)	ClinVar	Potentially affected PROSITE and ELM Motifs	Vps13 protein domain
Trp185Arg	101	-10.298	0.998	0.918	0.941	0.734	0.0	disease causing 0.999999867501632	no data	no data	no data	no data	-	-	LIG_PEX14_1, DOC_MAPK_GEN_1, LIG_ACTIN_WH2_2	-
Phe274Val	50	-1.732	0.844	0.752	0.412	0.134	0.0	disease causing 0.999987094277913	no data	no data	no data	no data	-	-	-	-
Gly567Glu	98	-1.501	0.015	0.263	0.264	0.070	0.0	disease causing 0.999724479670851	114	282342	0	0.0004442	Gly567Gly (2)	VUS	-	-
**Ala590Thr**	**58**	**-0.692**	**0.651**	**0.312**	**0.220**	**0.050**	**0.0**	**disease causing 0.99934312757362**	**236**	**282742**	**2**	**0.0008347**	**Ala590Glu (3) Ala590Val (14) Ala590Ala (31)**	**VUS**	**-**	**-**
**Ser824Ala**	**99**	**-0.520**	**0.079**	**0.159**	**0.244**	**0.062**	**0.0**	**polymorphism 0.999999999726318**	**45**	**282612**	**1**	**0.0001592**	**Ser824Phe (42) Ser824Ser (19)**	**VUS**	**-**	**-**
Thr1068Ile	89	-0.173	0.675	0.711	0.449	0.115	0.0	disease causing 0.999973855141115	2836	281306	35	0.01056	Thr1068Thr (6)	Benign	LIG_FHA_1, DOC_CYCLIN_1, (CLV_PCSK_SKI1_1), LIG_SUMO_SIM_PAR_1	-
Lys1129Arg	26	-0.948	0.937	0.162	0.450	0.101	0.0	disease causing 0.990735321628732	1303	282622	5	0.004610	Lys1129Glu (1)	Benign	-	-
Pro1133Ser	74	-1.692	0.374	0.586	0.539	0.134	0.0	disease causing 0.999999315450176	1	251222	0	0.000003981	Pro1133Thr (1)	LP	MOD_CK2_1, LIG_TRAF2_1, CK2_PHOSPHO_SITE	-
Asp1210Tyr	160	-6.983	0.999	0.922	0.928	0.525	0.0	disease causing 0.99999909362335	no data	no data	no data	no data	-	VUS	LIG_SH2_SRC, LIG_SH2_STAT5	-
Thr1289Ala	58	-1.544	0.991	0.343	0.215	0.048	0.0	disease causing 0.999926322700007	26	282234	0	0.00009212	Thr1289Ser (150)	VUS	-	-
Thr1289Ser	58	-0.827	0.991	0.216	0.162	0.030	0.0	disease causing 0.999995577038185	150	282188	1	0.0003788	Thr1289Ala (26)	VUS	-	-
**Ile1611Asn**	**194**	**-4.377**	**0.992**	**0.898**	**0.839**	**0.377**	**0.0**	**disease causing 0.999993993014875**	**no data**	**no data**	**no data**	**no data**	**Ile1611Ile (2)**	**P**	**(MOD_N-GLC_1), ASN_GLYCOSYLATION**	**-**
Lys1682Glu	56	-1.550	0.475	0.608	0.691	0.290	0.0	disease causing 0.999387572062778	1	282516	0	0.000003540	Lys1682Lys (1)	-	LIG_FHA_1, MOD_GSK_1, DOC_WW_Pin1_4, LIG_SH3_3, MOD_ProDKin_1, ELME000173	-
**Leu2168Arg**	**102**	**-3.350**	**0.814**	**0.789**	**0.793**	**0.307**	**0.0**	**disease causing 0.966354918683841**	**no data**	**no data**	**no data**	**no data**	**-**	**P**	**MOD_PKA_1, MOD_PKA_2, MOD_CK2_1, (CLV_NRD_NRD_1), LIG_ACTIN_WH2_2, MOD_NEK2_1, CAMP_PHOSPHO_SITE**	**-**
**Tyr2316Cys**	**194**	**-7.706**	**0.999**	**0.891**	**0.850**	**0.511**	**0.0**	**disease causing 0.999999164283618**	**2**	**282672**	**0**	**0.000007075**	**Tyr2316Tyr (2)**	**VUS**	**-**	**-**
Val2456Ile	29	-0.123	0.002	0.027	0.265	0.071	0.20	polymorphism 0.998023919296257	50	282710	0	0.0001768	-	Confl.	-	-
**Gly2620Asp[#]**	**94**	**-6.089**	**0.999**	**0.937**	**0.851**	**0.450**	**0.0**	**disease causing 0.999999996618357**	**no data**	**no data**	**no data**	**no data**	**-**	**Confl**	**MYRISTYL**	**SHR_BD (2607-2687)**
**Gly2704Arg**	**125**	**-6.956**	**1.000**	**0.877**	**0.931**	**0.764**	**0.0**	**disease causing 0.999999985985493**	**2**	**250782**	**0**	**0.000007975**	**-**	**VUS**	**(CLV_NRD_NRD_1), CLV_PCSK_KEX2_1**	**-**
**Ser2748Leu**	**145**	**-3.667**	**0.979**	**0.609**	**0.698**	**0.136**	**0.0**	**disease causing 0.999999504479773**	**1**	**250930**	**0**	**0.000003985**	**Ser2748Trp (1) Ser2748Ser (59)**	**VUS**	**ELME000173, DOC_MAPK_GEN_1, LIG_SUMO_SIM_PAR_1**	**-**
**Ile2795Thr**	**89**	**-1.678**	**0.801**	**0.344**	**0.608**	**0.163**	**0.0**	**disease causing 0.991215747760016**	**no data**	**no data**	**no data**	**no data**	**-**	**VUS**	**-**	**-**
Leu2821Ile	5	-1.311	0.892	0.297	0.364	0.101	0.0	disease causing 0.999758934712428	no data	no data	no data	no data	-	-	-	-
**Asn2968Ser**	**46**	**-4.611**	**0.994**	**0.871**	**0.635**	**0.215**	**0.0**	**disease causing 0.999998306414467**	**875**	**2282666**	**1**	**0.003096**	**-**	**Confl**	**(MOD_N-GLC_1), LIG_SUMO_SIM_PAR_1, ASN_GLYCOSYLATION**	**-**
Asn3088Tyr	143	-5.311	0.997	0.918	0.845	0.462	0.0	disease causing 0.999987671556758	no data	no data	no data	no data	Asn3088Asp (1)	-	LIG_FHA_1, MOD_N-GLC_1, LIG_PTB_Phospho_1, LIG_PTB_Apo_2, DOC_WW_Pin1-4, MOD_ProDKin_1, DOC_CKS1_1	-
Arg3198Trp	101	-1.729	0.409	0.139	0.656	0.267	0.0	polymorphism 0.999999999884398	1066	282148	4	0.003778	Arg3198Arg (1) Arg3198Gln (8)	Benign /LB	-	-
Ser3303Arg	110	-3.178	0.903	0.643	0.718	0.174	0.0	disease causing 0.999998165511474	no data	no data	no data	no data	-	-	MOD_GSK3_1, CLV_C14_CASPASE3-7	-
Val3420Met	21	-0.94	0.013	0.128	0.428	0.129	0.01	polymorphism 0.996794772703239	57	282858	0	0.000202	Val3445Val (1)	Confl	-	-
Thr3602Ile	89	-3.133	0.720	0.834	0.772	0.375	0.0	disease causing 0.999998165511474	2	251264	0	0.000007960	-	-	PKC_PHOSPHO_SITE	VPS13_C (3560-3706)
Ala3691Thr	58	-3.289	0.999	0.761	0.087	0.008	0.11	disease causing 0.999999998949835	643	253378	5	0.002538	Ala3691Asp (1)	Confl	LIG_FHA_1, MOD_GSK3_1, (MOD_N-GLC_1), MOD_NEK2_1, ASN_GLYCOSYLATION	VPS13_C (3560-3706)
Pro3962Arg	103	-2.366	0.101	0.101	0.719	0.213	0.0	disease causing 0.938688179688374	no data	no data	no data	no data	Pro3962Ala (247)	-	-	-

all cloned missense variants are in bold, [#]in combination with Asn2968Ser cloned, green indicates no negative impact, yellow indicates uncertain negative impact, red indicates potential negative impact

Supplementary Table T3: P value results of Dunnett's multiple comparisons test on Golgi ROI enrichment of overexpressed cloned VPS13B missense variants.

**Dunnett's multiple comparisons test**	**Mean Diff,**	**95,00% CI of diff,**	**Significant?**	**Summary**	**Adjusted P Value**	**Number of cells**
wt						**24**
wt vs. Ala590Thr	4,409	-0,6013 to 9,420	No	ns	0,1159	**28**
wt vs. Ser824Ala	1,617	-4,312 to 7,546	No	ns	0,9904	**15**
wt vs. Ile1611Asn	15,55	9,733 to 21,36	Yes	****	<0,0001	**16**
wt vs. Leu2168Arg	14,84	9,791 to 19,90	Yes	****	<0,0001	**27**
wt vs. Tyr2316Cys	15,18	8,395 to 21,95	Yes	****	<0,0001	**10**
wt vs. Gly2620Asp	16,07	10,75 to 21,39	Yes	****	<0,0001	**22**
wt vs. Gly2704Arg	15,01	9,755 to 20,27	Yes	****	<0,0001	**23**
wt vs. Ser2748Leu	15,50	9,446 to 21,56	Yes	****	<0,0001	**14**
wt vs. Ile2795Thr	2,076	-3,634 to 7,786	No	ns	0,9379	**17**
wt vs. Asn2968Ser	8,234	3,135 to 13,33	Yes	***	0,0001	**26**

Supplementary references

All supplementary references are cited in the main article.

1 Adzhubei IA, Schmidt S, Peshkin L, Ramensky VE, Gerasimova A, Bork P, Kondrashov AS, Sunyaev SR (2010) A method and server for predicting damaging missense mutations. Nat Methods 7: 248-249 Doi 10.1038/nmeth0410-248

2 Calabrese R, Capriotti E, Fariselli P, Martelli PL, Casadio R (2009) Functional annotations improve the predictive score of human disease-related mutations in proteins. Hum Mutat 30: 1237-1244 Doi 10.1002/humu.21047

3 Carss KJ, Arno G, Erwood M, Stephens J, Sanchis-Juan A, Hull S, Megy K, Grozeva D, Dewhurst E, Malka Set al (2017) Comprehensive Rare Variant Analysis via Whole-Genome Sequencing to Determine the Molecular Pathology of Inherited Retinal Disease. Am J Hum Genet 100: 75-90 Doi 10.1016/j.ajhg.2016.12.003

4 Chi ZH, Wei W, Bu DF, Li HH, Ding F, Zhu P (2018) Targeted high-throughput sequencing technique for the molecular diagnosis of primary immunodeficiency disorders. Medicine (Baltimore) 97: e12695 Doi 10.1097/MD.0000000000012695

5 Choi Y, Chan AP (2015) PROVEAN web server: a tool to predict the functional effect of amino acid substitutions and indels. Bioinformatics 31: 2745-2747 Doi 10.1093/bioinformatics/btv195

6 Choi Y, Sims GE, Murphy S, Miller JR, Chan AP (2012) Predicting the functional effect of amino acid substitutions and indels. PLoS One 7: e46688 Doi 10.1371/journal.pone.0046688

7 de Ligt J, Willemsen MH, van Bon BW, Kleefstra T, Yntema HG, Kroes T, Vulto-van Silfhout AT, Koolen DA, de Vries P, Gilissen Cet al (2012) Diagnostic exome sequencing in persons with severe intellectual disability. N Engl J Med 367: 1921-1929 Doi 10.1056/NEJMoa1206524

8 Dopazo J, Amadoz A, Bleda M, Garcia-Alonso L, Aleman A, Garcia-Garcia F, Rodriguez JA, Daub JT, Muntane G, Rueda Aet al (2016) 267 Spanish Exomes Reveal Population-Specific Differences in Disease-Related Genetic Variation. Mol Biol Evol 33: 1205-1218 Doi 10.1093/molbev/msw005

9 El Chehadeh S, Aral B, Gigot N, Thauvin-Robinet C, Donzel A, Delrue MA, Lacombe D, David A, Burglen L, Philip Net al (2010) Search for the best indicators for the presence of a VPS13B gene mutation and confirmation of diagnostic criteria in a series of 34 patients genotyped for suspected Cohen syndrome. J Med Genet 47: 549-553 Doi 10.1136/jmg.2009.075028

10 Falk MJ, Feiler HS, Neilson DE, Maxwell K, Lee JV, Segall SK, Robin NH, Wilhelmsen KC, Traskelin AL, Kolehmainen Jet al (2004) Cohen syndrome in the Ohio Amish. Am J Med Genet A 128A: 23-28 Doi 10.1002/ajmg.a.30033

11 Farwell KD, Shahmirzadi L, El-Khechen D, Powis Z, Chao EC, Tippin Davis B, Baxter RM, Zeng W, Mroske C, Parra MCet al (2015) Enhanced utility of family-centered diagnostic exome sequencing with inheritance model-based analysis: results from 500 unselected families with undiagnosed genetic conditions. Genet Med 17: 578-586 Doi 10.1038/gim.2014.154

12 Grantham R (1974) Amino acid difference formula to help explain protein evolution. Science 185: 862-864

13 Hennies HC, Rauch A, Seifert W, Schumi C, Moser E, Al-Taji E, Tariverdian G, Chrzanowska KH, Krajewska-Walasek M, Rajab Aet al (2004) Allelic heterogeneity in the COH1 gene explains clinical variability in Cohen syndrome. Am J Hum Genet 75: 138-145 Doi 10.1086/422219

14 Huang Z, Sun Y, Fan Y, Wang L, Liu H, Gong Z, Wang J, Yan H, Wang Y, Hu Get al (2018) Genetic Evaluation of 114 Chinese Short Stature Children in the Next Generation Era: a Single Center Study. Cell Physiol Biochem 49: 295-305 Doi 10.1159/000492879

15 Ionita-Laza I, Capanu M, De Rubeis S, McCallum K, Buxbaum JD (2014) Identification of rare causal variants in sequence-based studies: methods and applications to VPS13B, a gene involved in Cohen syndrome and autism. PLoS Genet 10: e1004729 Doi 10.1371/journal.pgen.1004729

16 Karczewski KJ, Francioli LC, Tiao G, Cummings BB, Alföldi J, Wang Q, Collins RL, Laricchia KM, Ganna A, Birnbaum DPet al (2019) Variation across 141,456 human exomes and genomes reveals the spectrum of loss-of-function intolerance across human protein-coding genes. bioRxiv: 531210 Doi 10.1101/531210

17 Katzaki E, Pescucci C, Uliana V, Papa FT, Ariani F, Meloni I, Priolo M, Selicorni A, Milani D, Fischetto Ret al (2007) Clinical and molecular characterization of Italian patients affected by Cohen syndrome. J Hum Genet 52: 1011-1017 Doi 10.1007/s10038-007-0208-4

18 Katzaki E, Pescucci C, Uliana V, Papa FT, Ariani F, Meloni I, Priolo M, Selicorni A, Milani D, Fischetto Ret al (2007) Clinical and molecular characterization of Italian patients affected by Cohen syndrome. J Hum Genet:

19 Kolehmainen J, Black GC, Saarinen A, Chandler K, Clayton-Smith J, Traskelin AL, Perveen R, Kivitie-Kallio S, Norio R, Warburg Met al (2003) Cohen syndrome is caused by mutations in a novel gene, COH1, encoding a transmembrane protein with a presumed role in vesicle-mediated sorting and intracellular protein transport. Am J Hum Genet 72: 1359-1369 Doi 10.1086/375454

20 Kolehmainen J, Wilkinson R, Lehesjoki AE, Chandler K, Kivitie-Kallio S, Clayton-Smith J, Traskelin AL, Waris L, Saarinen A, Khan Jet al (2004) Delineation of Cohen syndrome following a large-scale genotype-phenotype screen. Am J Hum Genet 75: 122-127 Doi 10.1086/422197

21 Koshimizu E, Miyatake S, Okamoto N, Nakashima M, Tsurusaki Y, Miyake N, Saitsu H, Matsumoto N (2013) Performance comparison of bench-top next generation sequencers using microdroplet PCR-based enrichment for targeted sequencing in patients with autism spectrum disorder. PLoS One 8: e74167 Doi 10.1371/journal.pone.0074167

22 Kumar P, Henikoff S, Ng PC (2009) Predicting the effects of coding non-synonymous variants on protein function using the SIFT algorithm. Nat Protoc 4: 1073-1081 Doi 10.1038/nprot.2009.86

23 Lee YK, Hwang SK, Lee SK, Yang JE, Kwak JH, Seo H, Ahn H, Lee YS, Kim J, Lim CSet al (2020) Cohen Syndrome Patient iPSC-Derived Neurospheres and Forebrain-Like Glutamatergic Neurons Reveal Reduced Proliferation of Neural Progenitor Cells and Altered Expression of Synapse Genes. J Clin Med 9: Doi 10.3390/jcm9061886

24 Li A, Gandhi A, Wang H, Traboulsi EI (2018) Bilateral angle closure glaucoma in a 28-year-old Cohen syndrome patient. Ophthalmic Genet 39: 657-658 Doi 10.1080/13816810.2018.1495746

25 Mi H, Muruganujan A, Thomas PD (2013) PANTHER in 2013: modeling the evolution of gene function, and other gene attributes, in the context of phylogenetic trees. Nucleic Acids Res 41: D377-386 Doi 10.1093/nar/gks1118

26 Mochida GH, Rajab A, Eyaid W, Lu A, Al-Nouri D, Kosaki K, Noruzinia M, Sarda P, Ishihara J, Bodell Aet al (2004) Broader geographical spectrum of Cohen syndrome due to COH1 mutations. J Med Genet 41: e87 Doi 10.1136/jmg.2003.014779

27 Ng PC, Henikoff S (2003) SIFT: Predicting amino acid changes that affect protein function. Nucleic Acids Res 31: 3812-3814

28 Pejaver V, Mooney SD, Radivojac P (2017) Missense variant pathogenicity predictors generalize well across a range of function-specific prediction challenges. Hum Mutat 38: 1092-1108 Doi 10.1002/humu.23258

29 Retterer K, Juusola J, Cho MT, Vitazka P, Millan F, Gibellini F, Vertino-Bell A, Smaoui N, Neidich J, Monaghan KGet al (2016) Clinical application of whole-exome sequencing across clinical indications. Genet Med 18: 696-704 Doi 10.1038/gim.2015.148

30 Rivera-Brugues N, Albrecht B, Wieczorek D, Schmidt H, Keller T, Gohring I, Ekici AB, Tzschach A, Garshasbi M, Franke Ket al (2011) Cohen syndrome diagnosis using whole genome arrays. J Med Genet 48: 136-140 Doi 10.1136/jmg.2010.082206

31 Schwarz JM, Cooper DN, Schuelke M, Seelow D (2014) MutationTaster2: mutation prediction for the deep-sequencing age. Nat Methods 11: 361-362 Doi 10.1038/nmeth.2890

32 Schwarz JM, Rodelsperger C, Schuelke M, Seelow D (2010) MutationTaster evaluates disease-causing potential of sequence alterations. Nat Methods 7: 575-576 Doi 10.1038/nmeth0810-575

33 Seifert W, Holder-Espinasse M, Kuhnisch J, Kahrizi K, Tzschach A, Garshasbi M, Najmabadi H, Walter Kuss A, Kress W, Laureys Get al (2009) Expanded mutational spectrum in Cohen syndrome, tissue expression, and transcript variants of COH1. Hum Mutat 30: E404-420 Doi 10.1002/humu.20886

34 Seifert W, Holder-Espinasse M, Spranger S, Hoeltzenbein M, Rossier E, Dollfus H, Lacombe D, Verloes A, Chrzanowska KH, Maegawa GHet al (2006) Mutational spectrum of COH1 and clinical heterogeneity in Cohen syndrome. J Med Genet 43: e22 Doi 10.1136/jmg.2005.039867

35 Stone EM, Andorf JL, Whitmore SS, DeLuca AP, Giacalone JC, Streb LM, Braun TA, Mullins RF, Scheetz TE, Sheffield VCet al (2017) Clinically Focused Molecular Investigation of 1000 Consecutive Families with Inherited Retinal Disease. Ophthalmology 124: 1314-1331 Doi 10.1016/j.ophtha.2017.04.008

36 Yang C, Hou M, Li Y, Sun D, Guo Y, Liu P, Liu Y, Song J, Zhang N, Wei Wet al (2018) Gene analysis: A rare gene disease of intellectual deficiency-Cohen syndrome. Int J Dev Neurosci 68: 83-88 Doi 10.1016/j.ijdevneu.2018.05.004

37 Yu TW, Chahrour MH, Coulter ME, Jiralerspong S, Okamura-Ikeda K, Ataman B, Schmitz-Abe K, Harmin DA, Adli M, Malik ANet al (2013) Using whole-exome sequencing to identify inherited causes of autism. Neuron 77: 259-273 Doi 10.1016/j.neuron.2012.11.002
